# Survey of the ants (Hymenoptera: Formicidae) of the Greek Thrace

**DOI:** 10.3897/BDJ.4.e7945

**Published:** 2016-04-13

**Authors:** Gregor Bračko, Kadri Kiran, Celal Karaman, Sebastian Salata, Lech Borowiec

**Affiliations:** ‡ University of Ljubljana, Ljubljana, Slovenia; § Trakya University, Edirne, Turkey; | University of Wrocław, Wrocław, Poland

## Introduction

The ant fauna of Greece has been an object of investigations for almost two centuries, but most of the contributions were restricted to particular parts or regions of the country. The first comprehensive checklist, comprising the past studies on Greek ants, was done only recently by Legakis (2011). In the last few years, the ant fauna of Greece was more intensively studied as part of the inventory of the ants of the Mediterranean region (Borowiec and Salata 2012, Borowiec and Salata 2013, Borowiec and Salata 2014b, Borowiec and Salata 2014a, Salata and Borowiec 2015a, Salata and Borowiec 2015b, Salata and Borowiec 2015c). Based on the above mentioned studies, it turned out that the Greek ant fauna is probably the richest in Europe, with about 280 recorded species including almost 20 endemic to this country. Among the geographic regions of Greece, Macedonia has richest ant fauna with at least 158 species recorded, followed by Dodecanese (111), Ionian Islands (107), East Aegean Islands (106), Peloponnese (102), Crete (98), Sterea Ellas (72), Thessaly (67), Cyclades (46), and Epirus (42) respectively (Borowiec & Salata unpublished data). The ants of Greek Thrace have been more or less neglected so far. This region has not been studied in recent years, while older data are very scarce. As a result, only 12 species were mentioned from this region (Legakis 2011).

Greek Thrace (or Western Thrace) is one of the geographic and historical regions of Greece. It is the eastern-most mainland part of the country, bordered by Greek Macedonia to the west, Bulgaria to the north (the southern part of Bulgaria is also known as the Bulgarian or Northern Thrace), Turkish (or Eastern) Thrace to the east and the Aegean Sea to the south. Most of the northern part of Greek Thrace is occupied by the Rhodope Mountains. Larger plains are situated especially in the south-western, central and north-eastern part of the region. A Mediterranean climate prevails in the southern part of Thrace and is modified by continental influences in the Rhodope Mountains (Encyclopædia Britannica 2015).

To improve the knowledge on Thracian ants, we conducted two field trips, in spring 2014 and in summer 2015, and included some previously collected unpublished material. Altogether, we compiled the samples from more than 70 localities throughout the region. As a result we present a check-list of all ant species recorded so far in Greek Thrace with comments on the taxonomy and distribution of poorly known or unnamed species.

## Materials and Methods

We sampled ants in spring 2014 and in summer 2015 from the sites in different parts of Greek Thrace. The main method, applied at all sites, was direct sampling (hand collecting). Ant nests and individual specimens were collected on the ground, in leaf litter, under stones, in dead wood, on tree trunks and twigs. This method was occasionally supplemented by litter sifting. Leaf litter from the ground was sieved into sifter with 1 x 1 cm wire mesh. Sieved material was placed on a white sheet and ants were collected.

**Figure 1.**
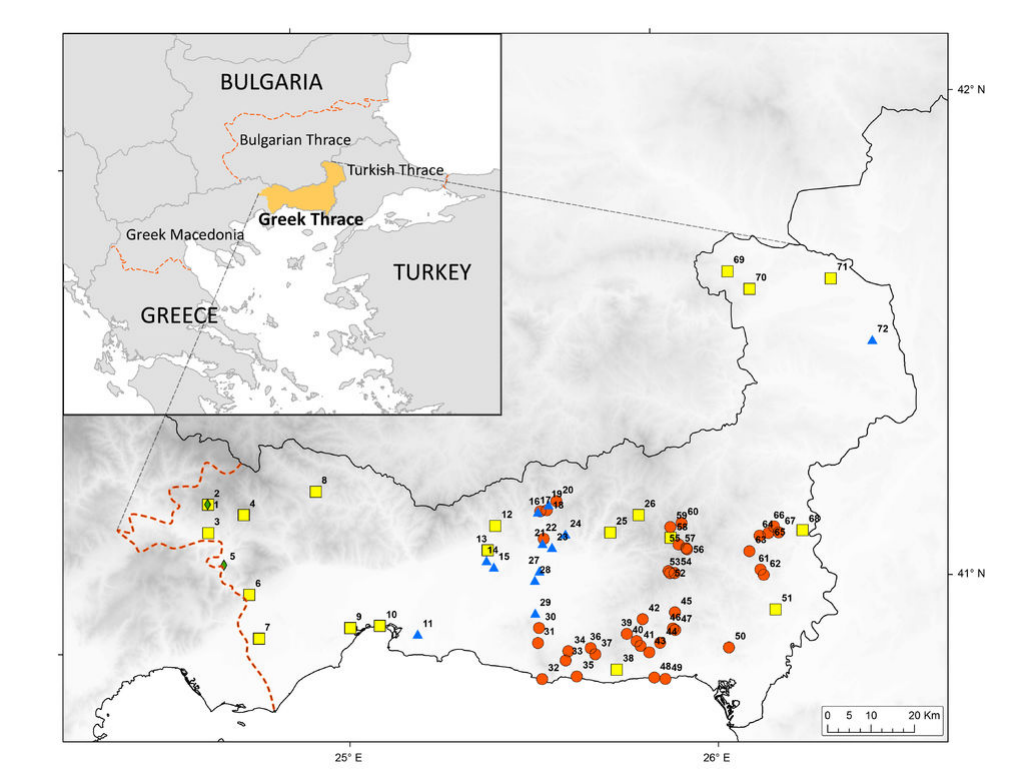
Sampling localities of ants in Greek Thrace (sampling in 1999 – green diamonds, in 2013 – blue triangles, in 2014 – yellow rectangles, in 2015 – red circles).

All specimens were preserved in 70-75% ethanol. Material sampled in 2014 is deposited in the personal collection of G. Bračko (Ljubljana, Slovenia) and in the collection of the Biological Department of Trakya University (Edirne, Turkey). Material sampled in 2015 is stored in the Department of Biodiversity and Evolutionary Taxonomy of the University of Wrocław (Wrocław, Poland). In this study we also included unpublished material collected in 2013 during general sampling of invertebrate fauna, deposited in the Biological Department of Trakya University. Finally, we examined ants deposited in the Natural History Museum of Crete (Heraklion, Greece), sampled in 1999. All sampled localities are described in Table 1 and shown in Fig. 1.

The following taxonomic literature was used for the identification of the collected ants: Agosti and Collingwood 1987a, Borowiec and Salata 2013, Csősz et al. (2007), Csősz et al. (2015), Karaman et al. (2011), Radchenko and Elmes (2010), Salata and Borowiec 2015b, Seifert (1992), Seifert (2003), Seifert (2007), Seifert (2012), Seifert and Schultz (2009). Where available, we compared our samples with high quality images of the type specimens on the AntWeb website (http://www.antweb.org). In the checklist all recently proposed nomenclatural changes made in the subfamily Myrmicinae Ward et al. 2015 and Formicinae Ward et al. 2016 were included.

Images of ant specimens shown in this paper were taken using a Nikon SMZ 1500 and Nikon SMZ 18 stereomicroscopes, Nikon D5200 photo camera and Helicon Focus software. All of them have assigned a CASENT number and are available on www.antweb.org.

Below, we present a list of all known ant species from Greek Thrace with the localities of the sampled material (see Table 1 for the description of locality codes) and literature data. We give information about the presence of the recorded species in other Greek regions, based on Borowiec and Salata (2012), Borowiec and Salata (2013), Borowiec and Salata (2014a), Borowiec and Salata unpubl. data, Csősz et al. (2015), as well as in the neighbouring regions of Bulgarian Thrace (Csősz et al. 2015, Lapeva-Gjonova and Kiran 2012, Lapeva-Gjonova et al. 2010) and Turkish Thrace (Csősz et al. 2015, Kiran and Karaman 2012, Kiran and Karaman unpubl. data). We add comments on the taxonomy and distribution of poorly known or unnamed species. Following the list of recorded species, we give notes on the taxa which were mentioned in Legakis (2011), but their occurence in Greek Thrace is doubtful.

## Results

### List of ants of Greek Thrace

*Aphaenogaster epirotes* (Emery, 1895)

Records in Greek Thrace: 9, 13, 25, 51, 70

Distribution in Greece and neighbouring regions: East Aegean Is., Ionian Is., Macedonia, Peloponnese, Sterea Ellas; Bulgarian Thrace, Turkish Thrace

**Figure 2.**
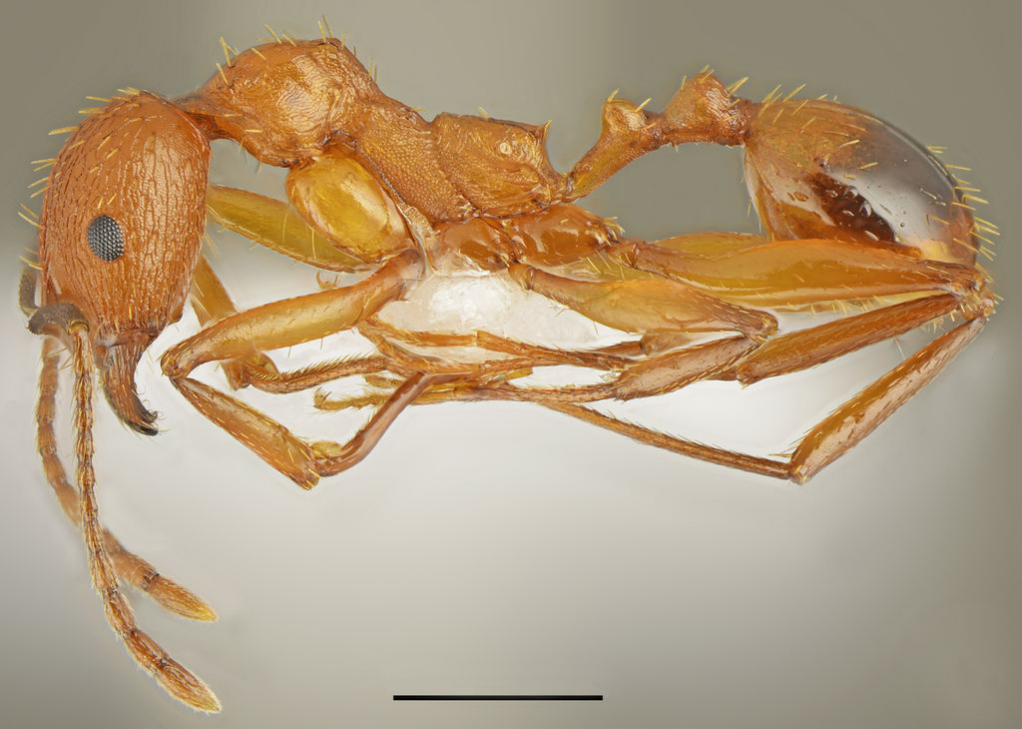
*Aphaenogaster festae* worker (specimen code: CASENT0763854). Lateral view of the body (scale bar = 1mm).

*Aphaenogaster festae* Emery, 1915 (Fig. 2)

Records in Greek Thrace: 6, 30; Legakis 2011

Distribution in Greece and neighbouring regions: Dodecanese, East Aegean Is., Epirus, Macedonia

*Aphaenogaster subterranea* (Latreille, 1798)

Records in Greek Thrace: 5, 8, 18, 36, 41, 43, 44, 45, 46, 47, 51, 52, 53, 54, 56, 57, 59, 60, 61, 62, 63, 65, 66, 67, 70

Distribution in Greece and neighbouring regions: Cyclades, East Aegean Is., Epirus, Ionian Is., Macedonia, Peloponnese, Sterea Ellas, Thessaly; Bulgarian Thrace, Turkish Thrace

**Figure 3.**
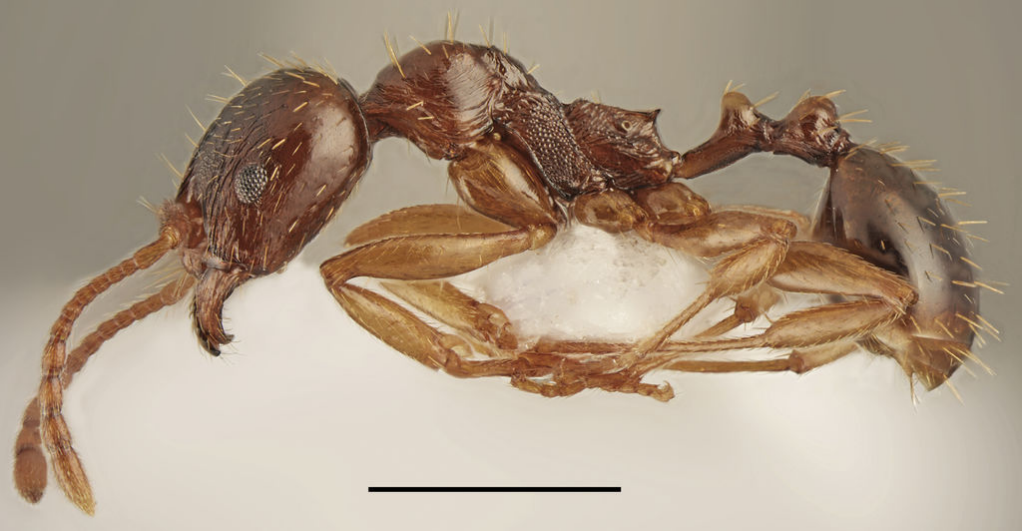
Aphaenogaster cf. subterranea worker (specimen code: CASENT0763853). Lateral view of the body (scale bar = 1mm).

Aphaenogaster cf. subterranea (Fig. 3)

Records in Greek Thrace: 62

Notes: Our material from Greece showed that at least seven morphospecies belonging to *A. subterranea* complex occur in this country. However, only two of them have a formal name, i.e. *A. subterranea* (Latreille, 1798) and *A. lesbica* Forel, 1913. Specimens sampled in the vicinity of Lefkimmi (62) look very similar to samples of *A. lesbica*, recorded hitherto only from Lesbos and to another unnamed morphospecies, spreaded in various localities of Pieria Mountains in southern part of Macedonia. This complex is now under revision and status of the sample from Thrace will be explained in the future.

*Bothriomyrmex communistus* Santschi, 1919

Records in Greek Thrace: 51, 58

Distribution in Greece and neighbouring regions: Dodecanese, Eastern Aegean Is., Epirus, Ionian Is., Macedonia, Peloponnese, Sterea Ellas, Thessaly; Bulgarian Thrace, Turkish Thrace

*Bothriomyrmex corsicus* Santschi, 1923

Records in Greek Thrace: 69

Distribution in Greece and neighbouring regions: East Aegean Is., Ionian Is., Macedonia, Peloponnese; Bulgarian Thrace, Turkish Thrace

*Camponotus aegaeus* Emery, 1915

Records in Greek Thrace: 66, 68

Distribution in Greece and neighbouring regions: Crete, Dodecanese, East Aegean Is., Macedonia; Turkish Thrace

*Camponotus aethiops* (Latreille, 1798)

Records in Greek Thrace: 3, 5, 7, 9, 12, 13, 25, 30, 31, 32, 33, 38, 48, 51, 53, 58, 63, 66, 68, 69, 70; Legakis 2011 [as *Camponotus marginatus* (Latreille, 1798)]

Distribution in Greece and neighbouring regions: Crete, Cyclades, Dodecanese, East Aegean Is., Epirus, Ionian Is., Macedonia, Peloponnese, Sterea Ellas, Thessaly; Bulgarian Thrace, Turkish Thrace

*Camponotus atricolor* (Nylander, 1849)

Records in Greek Thrace: 3, 7, 51, 69, 71

Distribution in Greece and neighbouring regions: Dodecanese, East Aegean Is., Macedonia, Peloponnese; Turkish Thrace

*Camponotus dalmaticus* (Nylander, 1849)

Records in Greek Thrace: 30, 35, 36, 41, 44, 46, 61, 69

Distribution in Greece and neighbouring regions: East Aegean Is., Ionian Is., Macedonia, Peloponnese, Sterea Ellas, Thessaly; Bulgarian Thrace, Turkish Thrace

*Camponotus fallax* (Nylander, 1856)

Records in Greek Thrace: 20, 71

Distribution in Greece and neighbouring regions: East Aegean Is., Ionian Is., Macedonia, Peloponnese; Bulgarian Thrace, Turkish Thrace

*Camponotus gestroi* Emery, 1878

Records in Greek Thrace: 13, 35, 38

Distribution in Greece and neighbouring regions: Crete, Cyclades, Dodecanese, East Aegean Is., Ionian Is., Macedonia, Peloponnese; Bulgarian Thrace, Turkish Thrace

*Camponotus ionius* Emery, 1920

Records in Greek Thrace: 6

Distribution in Greece and neighbouring regions: Cyclades, Dodecanese, East Aegean Is., Ionian Is., Macedonia, Peloponnese, Sterea Ellas, Thessaly

*Camponotus kiesenwetteri* (Roger, 1859)

Records in Greek Thrace: 38

Distribution in Greece and neighbouring regions: Crete, Cyclades, Dodecanese, East Aegean Is., Ionian Is., Macedonia, Peloponnese, Sterea Ellas

*Camponotus lateralis* (Olivier, 1792)

Records in Greek Thrace: 10, 12, 17, 18, 20, 30, 31, 33, 35, 36, 37, 41, 42, 43, 44, 45, 46, 47, 48, 50, 51, 61, 62, 63, 64, 65, 66, 67, 69, 71

Distribution in Greece and neighbouring regions: Crete, Cyclades, Dodecanese, East Aegean Is., Epirus, Ionian Is., Macedonia, Peloponnese, Sterea Ellas, Thessaly; Bulgarian Thrace, Turkish Thrace

*Camponotus oertzeni* Forel, 1889 (Fig. 4)

Records in Greek Thrace: 4, 8, 17, 25, 26, 36, 46, 56, 60, 61, 63, 64, 65, 66

Distribution in Greece and neighbouring regions: Dodecanese, East Aegean Is., Epirus, Ionian Is., Macedonia

Notes: This poorly known species was recorded from five Greek regions (Borowiec and Salata 2012, Legakis 2011). Our material from the Balkan Peninsula shows that *C. oertzeni* is more common and widespread as indicated from the checklists, probably due to the misidentification with very common *C. aethiops*. Its redescription, habitat preferences and remarks on the diagnostic characters in comparison with *C. aethiops* will be provided in a separate paper (Salata & Borowiec in preparation).

*Camponotus piceus* (Leach, 1825)

Records in Greek Thrace: 4, 17, 25, 46, 69, 70

Distribution in Greece and neighbouring regions: Crete, Cyclades, Dodecanese, East Aegean Is., Epirus, Ionian Is., Macedonia, Peloponnese, Sterea Ellas, Thessaly; Bulgarian Thrace, Turkish Thrace

*Camponotus samius* Forel, 1889

Records in Greek Thrace: 12, 34, 46, 69, 70

Distribution in Greece and neighbouring regions: Cyclades, Dodecanese, East Aegean Is., Macedonia, Peloponnese, Sterea Ellas; Bulgarian Thrace, Turkish Thrace

*Camponotus vagus* (Scopoli, 1763)

Records in Greek Thrace: 20, 53, 56

Distribution in Greece and neighbouring regions: East Aegean Is., Epirus, Ionian Is., Macedonia, Peloponnese, Sterea Ellas, Thessaly; Bulgarian Thrace, Turkish Thrace

*Cardiocondyla bulgarica* Forel, 1892

Records in Greek Thrace: 42, 71

Distribution in Greece and neighbouring regions: Dodecanese, East Aegean Is., Macedonia; Bulgarian Thrace, Turkish Thrace

*Cataglyphis nodus* (Brullé, 1833)

Records in Greek Thrace: 3, 7, 9, 11, 16, 17, 18, 20, 30, 31, 32, 35, 41, 43, 46, 48, 52, 61, 66, 69, 71

Distribution in Greece and neighbouring regions: Crete, Dodecanese, East Aegean Is., Epirus, Ionian Is., Macedonia, Peloponnese, Sterea Ellas, Thessaly; Bulgarian Thrace, Turkish Thrace

*Cataglyphis viaticoides* (André, 1881) (Fig. 5​)

Records in Greek Thrace: 38

Distribution in Greece and neighbouring regions: East Aegean Is.; Turkish Thrace

Notes: The status of this species was misinterpreted probably due to confusion with type material preserved in the Museum of Natural History in Paris. André (1881), in the original description, clearly wrote that specimens named as a Myrmecocystus albicans var. viaticoides were collected in Beyruth (Lebanon). As diagnostic features he noted red colouration of the head and mesosoma and mostly black gaster. In the same paper he described another taxon: Myrmecocystus albicans var. lividus. Specimens of this species were collected in Jaffa, Syria (now Israel) and were distinguished by whole body pale reddish and only apex of gaster infuscate (now dried syntypes appear faded and are almost completely yellow). Surprisingly, in the material preserved in Paris Museum one bicoloured syntype with dark gaster with determination label “*viaticoides*” has locality label “Syrie” (available in AntWeb https://www.antweb.org/specimen/CASENT0912236) and another one, uniformly yellow syntype with determination label “*viaticoides*”, has locality label “Beyrouth” (available in AntWebhttps://www.antweb.org/specimen/CASENT0915503). In the same collection there is also one syntype of uniformly yellow body with determination label “*lividus*” and locality label “Syrie” (available in AntWebhttps://www.antweb.org/specimen/CASENT0915499). We found two other syntypes with determination label “*lividus*” and locality label “Jaffa” in Forel’s collection in Genève (available in AntWeb https://www.antweb.org/specimen/CASENT0911099) and in Santschi’s collection in Basel (available in AntWeb https://www.antweb.org/specimen/CASENT0912207). Radchenko (1997) studied syntype labelled “Beyrouth” (with mostly yellow abdomen) and suggested that records of bicoloured *Cataglyphis viaticoides* from Turkey, Caucasus and Iran concern *Cataglyphis rubra* (Forel, 1903). In his next paper with a key to Asian members of the genus *Cataglyphis* (Radchenko 1998), he named bicoloured taxon as a *C. rubra* and unicoloured taxa as a *C. lividus* and *C. vi* aticoides with note that *C. viaticoides* is a problematic species. Agosti (1990), in his review of *Cataglyphis*, noted that syntypes of *C. viaticoides* do not correspond with species description but he did not propose any solution of this problem. In our opinion only syntypes from Beyruth should be the true types of *C. viaticoides*, while syntypes from Syrie or Jaffa should be treated as a true types of *C. lividus*. Probably, discussed above syntypes, were inversely labeled in Paris Museum (bicolored specimens should have label "Beyrouth / Abeille" and uniformly yellow specimens should have label “Jaffa / Abeille” or "Syrie / Abeille"). *Cataglyphis viaticoides* is the only species of the mentioned above two taxa which occurs in Greece. Data on the distribution of *C. bicolor* (Fabricius, 1793) in Transcaucasia, Asia Minor, Iran, the Middle East and Arabian Peninsula should refer to *C. viaticoides*. True *C. bicolor* is restricted only to North Africa (Wehner et al. 1994, C. Galkowski pers. comm).

*Colobopsis truncata* (Spinola, 1808)

Records in Greek Thrace: 7, 10, 71

Distribution in Greece and neighbouring regions: Crete, Dodecanese, East Aegean Is., Ionian Is., Macedonia, Peloponnese, Sterea Ellas, Thessaly; Bulgarian Thrace, Turkish Thrace

*Crematogaster ionia* Forel, 1911

Records in Greek Thrace: 6

Distribution in Greece and neighbouring regions: Crete, Cyclades, Dodecanese, East Aegean Is., Epirus, Ionian Is., Macedonia, Peloponnese, Sterea Ellas, Thessaly; Turkish Thrace

*Crematogaster lorteti* Forel, 1910

Records in Greek Thrace: 25, 51, 69

Distribution in Greece and neighbouring regions: East Aegean Is., Macedonia, Sterea Ellas; Turkish Thrace

*Crematogaster schmidti* (Mayr, 1853)

Records in Greek Thrace: 3, 5, 9, 10, 12, 17, 18, 20, 30, 31, 33, 34, 35, 36, 37, 40, 41, 42, 43, 44, 45, 46, 47, 48, 50, 51, 54, 61, 62, 63, 65, 66, 68, 69, 70, 71

Distribution in Greece and neighbouring regions: Crete, Cyclades, Dodecanese, East Aegean Is., Ionian Is., Macedonia, Peloponnese, Sterea Ellas, Thessaly; Bulgarian Thrace, Turkish Thrace

*Crematogaster sordidula* (Nylander, 1849)

Records in Greek Thrace: 6, 9, 12, 38, 46, 69, 70; Legakis 2011 [as *Crematogaster mayri* (Nylander, 1849)]

Distribution in Greece and neighbouring regions: Crete, Cyclades, Dodecanese, East Aegean Is., Ionian Is., Macedonia, Peloponnese, Sterea Ellas, Thessaly; Bulgarian Thrace, Turkish Thrace

*Dolichoderus quadripunctatus* (Linnaeus, 1771)

Records in Greek Thrace: 18, 20, 35, 47, 65

Distribution in Greece and neighbouring regions: East Aegean Is., Epirus, Ionian Is., Macedonia, Peloponnese, Thessaly; Bulgarian Thrace, Turkish Thrace

*Formica cinerea* Mayr, 1853

Records in Greek Thrace: 5

Distribution in Greece and neighbouring regions: Epirus, Macedonia, Thessaly; Bulgarian Thrace, Turkish Thrace

*Formica clara* Forel, 1886

Records in Greek Thrace: 7, 10, 15, 16, 22, 27, 28, 29, 42, 48, 49, 53, 62, 71, 72

Distribution in Greece and neighbouring regions: East Aegean Is., Macedonia, Peloponnese; Bulgarian Thrace, Turkish Thrace

*Formica cunicularia* Latreille, 1798

Records in Greek Thrace: 1, 2, 5, 42, 46, 47, 49, 53, 58, 59, 60

Distribution in Greece and neighbouring regions: Crete, East Aegean Is., Epirus, Macedonia, Sterea Ellas; Bulgarian Thrace, Turkish Thrace

*Formica fusca* Linnaeus, 1758

Records in Greek Thrace: 5, 56, 60

Distribution in Greece and neighbouring regions: Epirus, Ionian Is., Macedonia, Peloponnese; Bulgarian Thrace, Turkish Thrace

*Formica gagates* Latreille, 1798

Records in Greek Thrace: 5, 26, 40, 53, 55, 65

Distribution in Greece and neighbouring regions: Epirus, Ionian Is., Macedonia, Sterea Ellas, Thessaly; Bulgarian Thrace, Turkish Thrace

*Formica pratensis* Retzius, 1783

Records in Greek Thrace: 1, 2, 7

Distribution in Greece and neighbouring regions: Macedonia; Bulgarian Thrace, Turkish Thrace

*Formica rufa* Linnaeus, 1761

Records in Greek Thrace: 56; Legakis 2011

Distribution in Greece and neighbouring regions: Macedonia; Bulgarian Thrace

*Formica rufibarbis* Fabricius, 1793

Records in Greek Thrace: 1, 5

Distribution in Greece and neighbouring regions: Cyclades, East Aegean Is., Epirus, Macedonia, Peloponnese, Sterea Ellas, Thessaly; Bulgarian Thrace, Turkish Thrace

*Formica sanguinea* Latreille, 1798

Records in Greek Thrace: 1, 5, 53

Distribution in Greece and neighbouring regions: Macedonia, Peloponnese; Bulgarian Thrace, Turkish Thrace

*Lasius alienus* (Förster, 1850)

Records in Greek Thrace: 1, 2, 8, 19, 26, 33, 34, 35, 36, 37, 42, 43, 46, 47, 58, 60, 66, 67, 70

Distribution in Greece and neighbouring regions: Cyclades, East Aegean Is., Epirus, Macedonia, Peloponnese, Sterea Ellas, Thessaly; Bulgarian Thrace, Turkish Thrace

*Lasius balcanicus* Seifert, 1988 or *L. distinguendus* (Emery, 1916)

Records in Greek Thrace: 1, 69; Legakis 2011 [as *L. distinguendus*]

Distribution in Greece and neighbouring regions: Macedonia; Bulgarian Thrace, Turkish Thrace

Notes: Proper identification of both species requires nest samples with gynes (Seifert 2007). We have only workers available in our material.

*Lasius brunneus* (Latreille, 1798)

Records in Greek Thrace: 26, 47, 50, 53, 54, 59

Distribution in Greece and neighbouring regions: East Aegean Is., Ionian Is., Macedonia, Peloponnese, Sterea Ellas, Thessaly; Bulgarian Thrace, Turkish Thrace

*Lasius flavus* (Fabricius, 1782)

Records in Greek Thrace: 42

Distribution in Greece and neighbouring regions: East Aegean Is., Epirus, Ionian Is., Macedonia, Peloponnese, Sterea Ellas, Thessaly; Bulgarian Thrace, Turkish Thrace

*Lasius fuliginosus* (Latreille, 1798)

Records in Greek Thrace: 1, 52, 69, 71

Distribution in Greece and neighbouring regions: Macedonia; Bulgarian Thrace, Turkish Thrace

*Lasius illyricus* Zimmermann, 1935

Records in Greek Thrace: 4, 5, 8, 20, 26, 52, 53, 54, 55, 56, 58, 59, 60

Distribution in Greece and neighbouring regions: Crete, Ionian Is., Macedonia, Peloponnese

*Lasius jensi* Seifert, 1982

Records in Greek Thrace: 4

Distribution in Greece and neighbouring regions: Macedonia; Bulgarian Thrace

*Lasius lasioides* (Emery, 1869)

Records in Greek Thrace: 48

Distribution in Greece and neighbouring regions: Crete, Dodecanese, Ionian Is., Macedonia, Thessaly; Turkish Thrace

*Lasius myops* Forel, 1894

Records in Greek Thrace: 44

Distribution in Greece and neighbouring regions: Macedonia; Turkish Thrace

*Lasius neglectus*/*turcicus* complex

Records in Greek Thrace: 6, 12, 13, 18, 20, 22, 30, 32, 38, 42, 45, 47, 48, 49, 50, 51, 56, 65, 68, 69

Distribution in Greece and neighbouring regions: Crete, Dodecanese, East Aegean Is., Macedonia, Peloponnese, Sterea Ellas; Turkish Thrace

Notes: The status of both taxa, *L. neglectus* Van Loon, Boomsma & Andrasfalvy, 1990 and *L. turcicus* Santschi, 1921, is still under discussion. Populations of both taxa show differences in biology and ecology and quite expressed morphometric differences in males (these are less expressed in female castes), which could indicate that these are two distinct species (Seifert 2000). However, preliminary molecular studies suggest conspecifity of both taxa, what confirm hypothesis of occurrence of two eco-morphotypes of one species.

*Lasius niger* (Linnaeus, 1758)

Records in Greek Thrace: 71

Distribution in Greece and neighbouring regions: Macedonia (see notes below); Bulgarian Thrace, Turkish Thrace

Notes: *Lasius niger* was listed for six Greek regions (Legakis 2011), but most records are from the period before the revision of *Lasius* s. str. (Seifert 1992) and probably concern other similar species. In recently collected Greek material, we have only one reliable record from Greek Macedonia (Borowiec and Salata 2012).

*Lasius paralienus* Seifert, 1992

Records in Greek Thrace: 3, 5, 55

Distribution in Greece and neighbouring regions: Crete, Dodecanese, East Aegean Is., Ionian Is., Macedonia, Peloponnese, Thessaly; Bulgarian Thrace, Turkish Thrace

*Lepisiota frauenfeldi* (Mayr, 1855)

Records in Greek Thrace: 6, 12, 13, 18, 20, 22, 24, 25, 29, 30, 32, 34, 35, 43, 44, 46, 47, 48, 49, 50, 67, 68

Distribution in Greece and neighbouring regions: Crete, Cyclades, Dodecanese, East Aegean Is., Ionian Is., Macedonia, Peloponnese, Sterea Ellas, Thessaly; Bulgarian Thrace, Turkish Thrace

*Leptothorax acervorum* (Fabricius, 1793)

Records in Greek Thrace: 1

Distribution in Greece and neighbouring regions: Macedonia (see notes below); Bulgarian Thrace, Turkish Thrace

Notes: *Leptothorax acervorum* was only recorded generally from Greece by Agosti and Collingwood (1987b). In recently collected material we found this species only from two localities in Drama regional unit in Macedonia.

*Liometopum microcephalum* (Panzer, 1798)

Records in Greek Thrace: 6, 16, 17, 24, 25, 42, 47

Distribution in Greece and neighbouring regions: East Aegean Is., Epirus, Ionian Is., Macedonia, Peloponnese, Sterea Ellas, Thessaly; Bulgarian Thrace, Turkish Thrace

**Figure 6.**
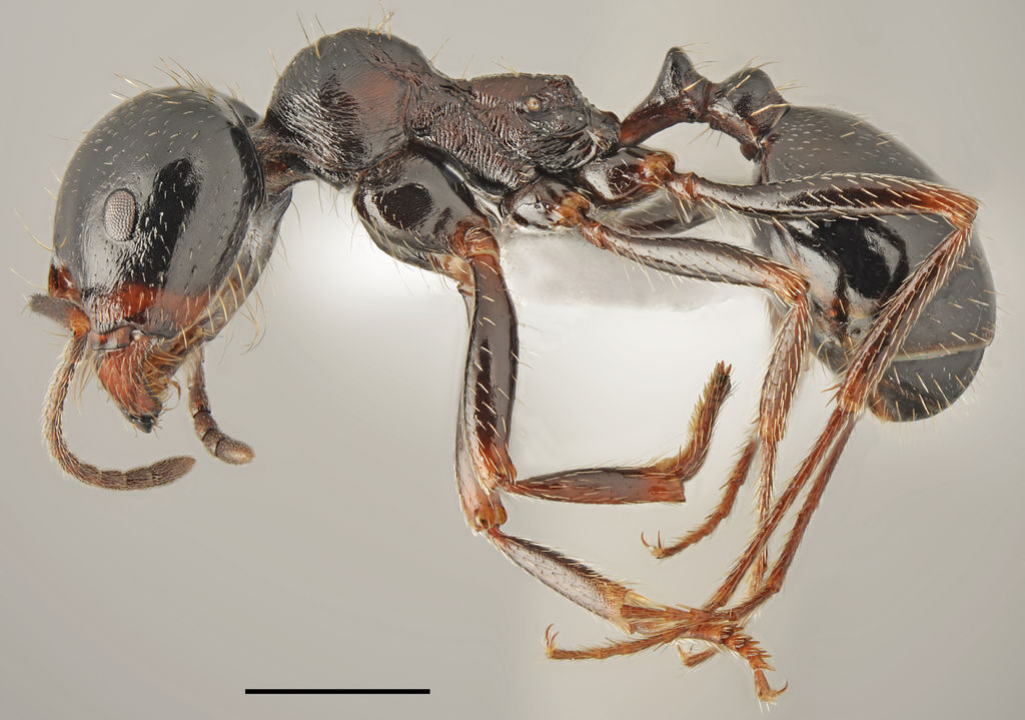
Messor cf. ebeninus worker (specimen code: CASENT0763589). Lateral view of the body (scale bar = 1mm).

Messor cf. ebeninus (Fig. 6​)

Records in Greek Thrace: 7, 12, 25, 26

Distribution in Greece and neighbouring regions: Turkish Thrace

Notes: Our samples named here as Messor cf. ebeninus and M. cf. semirufus (listed below) belong to the *Messor semirufus* complex. This complex comprises numerous names of various rank, partly available to nomenclature. Most taxa were described from the eastern part of the Mediterranean Basin (Borowiec 2014, Tohmé and Tohmé 1981). Our material from Greece suggests the occurrence of at least three morphospecies of complex in this country but their correct identification will be possible only after the revision of all names proposed in this group.

*Messor hellenius* Agosti & Collingwood, 1987

Records in Greek Thrace: 3, 6, 7, 10, 15, 19, 21, 22, 24, 27, 29, 48, 68, 71

Distribution in Greece and neighbouring regions: Cyclades, Dodecanese, East Aegean Is., Macedonia, Peloponnese, Sterea Ellas, Thessaly; Turkish Thrace

*Messor oertzeni* Forel, 1910

Records in Greek Thrace: 9, 30, 32, 33, 44, 48; Legakis 2011

Distribution in Greece and neighbouring regions: East Aegean Is., Macedonia, Thrace; Bulgarian Thrace, Turkish Thrace

*Messor orientalis* (Emery, 1898)

Records in Greek Thrace: 5, 9, 13, 17, 25, 30, 33, 35, 43, 48, 49, 58

Distribution in Greece and neighbouring regions: Crete, Dodecanese, East Aegean Is., Macedonia, Peloponnese, Sterea Ellas, Thessaly; Turkish Thrace

Messor cf. semirufus

Records in Greek Thrace: 3

Distribution in Greece and neighbouring regions: Turkish Thrace

Notes: See notes under M. cf. ebeninus.

Messor cf. structor

Records in Greek Thrace: 2, 23, 27, 29, 50, 53, 72

Distribution in Greece and neighbouring regions: Crete, Cyclades, Dodecanese, East Aegean Is., Ionian Is., Macedonia, Peloponnese, Sterea Ellas; Bulgarian Thrace, Turkish Thrace

Notes: According to molecular studies, taxon previously named as a *Messor structor* (Latreille, 1798) comprises two cryptic species. Both can be found in different parts of the Balkan Peninsula, also in southern Bulgaria close to Greek border (Schlick-Steiner et al. 2006b). Since many available names of various rank were proposed in the *Messor structor* complex, the proper identification of our samples is impossible prior to the revision of all proposed taxa.

*Messor wasmanni* Krausse, 1910

Records in Greek Thrace: 9, 13, 15, 29, 30, 32, 33, 38, 47, 48, 49, 67, 68, 71

Distribution in Greece and neighbouring regions: Crete, Cyclades, Dodecanese, East Aegean Is., Ionian Is., Macedonia, Peloponnese, Sterea Ellas, Thessaly; Bulgarian Thrace, Turkish Thrace

*Myrmecina graminicola* (Latreille, 1802)

Records in Greek Thrace: 5, 6, 9, 60, 70, 71

Distribution in Greece and neighbouring regions: Crete, Cyclades, Dodecanese, Ionian Is., Macedonia, Peloponnese, Sterea Ellas, Thessaly; Bulgarian Thrace, Turkish Thrace

*Myrmica hellenica* Finzi, 1926

Records in Greek Thrace: 7, 10

Distribution in Greece and neighbouring regions: Ionian Is., Macedonia, Peloponnese; Bulgarian Thrace, Turkish Thrace

*Myrmica lonae* Finzi, 1926

Records in Greek Thrace: 69

Distribution in Greece and neighbouring regions: Macedonia; Bulgarian Thrace, Turkish Thrace

*Myrmica sabuleti* Meinert, 1861

Records in Greek Thrace: 1, 55, 59, 60

Distribution in Greece and neighbouring regions: Dodecanese, Epirus, Macedonia, Sterea Ellas; Bulgarian Thrace, Turkish Thrace

*Myrmica scabrinodis* Nylander, 1846

Records in Greek Thrace: 1, 5

Distribution in Greece and neighbouring regions: Ionian Is., Macedonia; Bulgarian Thrace, Turkish Thrace

*Myrmica specioides* Bondroit, 1918

Records in Greek Thrace: 71

Distribution in Greece and neighbouring regions: Macedonia; Bulgarian Thrace, Turkish Thrace

Pheidole cf. pallidula

Records in Greek Thrace: 3, 5, 6, 8, 9, 12, 14, 17, 18, 20, 22, 26, 30, 32, 33, 35, 36, 37, 38, 41, 42, 43, 46, 47, 48, 50, 51, 54, 64, 67, 68, 69, 70

Distribution in Greece and neighbouring regions: Crete, Cyclades, Dodecanese, East Aegean Is., Epirus, Ionian Is., Macedonia, Peloponnese, Sterea Ellas, Thessaly; Bulgarian Thrace, Turkish Thrace

Notes: Mediterranean populations of taxon named *Pheidole pallidula* (Nylander, 1849) are now under revision (B. Seifert pers. comm.). Preliminary results suggest occurrence of at least three taxa in the Mediterranean Basin. True *P. pallidula* is restricted mostly to the western part of the studied area, while in Greece probably occur two other species although introduction of true *P. pallidula* to tourist resorts is also possible.

*Plagiolepis pallescens* sensu Radchenko (Fig. 7)

Records in Greek Thrace: 48, 49, 67

Distribution in Greece and neighbouring regions: Crete, Cyclades, Dodecanese, East Aegean Is., Epirus, Ionian Is., Macedonia, Peloponnese, Sterea Ellas, Thessaly; Turkish Thrace

Notes: Under this taxon we placed the samples of *Plagiolepis* with densely pubescent first gaster tergite. Radchenko (1996) reviewed members of the genus *Plagiolepis* from Central and Southern Palaearctic and proposed the name *P. pallescens* Forel, 1889 for taxon with densely pubescent first gaster tergite of both workers and gynes. We studied syntype of *P. pallescens* described from Rhodes Island preserved in Museum of Genève (available in AntWeb https://www.antweb.org/specimen/CASENT0909854) which is yellowish and has first gaster tergite sparsely pubescent. We have many samples of yellow coloured and sparsely pubescent mature workers collected from Rhodes and observed nests with workers of exclusively yellow aberration and nests with mixed yellow and brown aberrations. Both light and dark colored specimens showed similar level of sclerotization of cuticle. Thus the light colour in this case is not indicative of callow workers. Our specimens of *Plagiolepis pallescens* sensu Radchenko have darker colouration, from yellowish brown to brown. Only the callow workers are lighter yellowish. In morphometric characters samples of both yellow and dark aberrations from Rhodes appear to be conspecific with *Plagiolepis taurica* Santschi, 1920, a sparsely pubescent species which is also variable in colour (Salata and Borowiec in preparation). *Plagiolepis schmitzi* Forel, 1885 is the only other densely pubescent taxon from the Mediterranean area, distributed from Portugal to Sicily (recent record from Iran by Ghahari et al. 2015 is probably based on misidentification), but it is not conspecific with the eastern form named by Radchenko 1996 as *P. pallescens*. Therefore, densely pubescent taxon from the eastern part of the Mediterranean Basin has no valid name and its proper identification needs a revision of several available names of infraspecific taxa proposed from the Mediterranean area.

*Plagiolepis pygmaea* (Latreille, 1798)

Records in Greek Thrace: 6, 8, 9, 12, 17, 20, 30, 32, 35, 36, 37, 41, 42, 43, 44, 45, 46, 47, 50, 51, 61, 64, 65, 66, 69, 70

Distribution in Greece and neighbouring regions: Crete, Cyclades, Dodecanese, East Aegean Is., Epirus, Ionian Is., Macedonia, Peloponnese, Sterea Ellas, Thessaly; Bulgarian Thrace, Turkish Thrace

*Plagiolepis taurica* Santschi, 1920

Records in Greek Thrace: 2, 4, 6, 22, 38, 58, 68

Distribution in Greece and neighbouring regions: Crete, Cyclades, Dodecanese, East Aegean Is., Ionian Is., Macedonia, Peloponnese; Bulgarian Thrace, Turkish Thrace

*Ponera coarctata* (Latreille, 1802)

Records in Greek Thrace: 30, 60, 71

Distribution in Greece and neighbouring regions: Epirus, Ionian Is., Macedonia, Sterea Ellas, Peloponnese, Thessaly; Bulgarian Thrace, Turkish Thrace

*Ponera testacea* Emery, 1895

Records in Greek Thrace: 46, 69, 70

Distribution in Greece and neighbouring regions: Crete, Ionian Is., Macedonia, Peloponnese; Bulgarian Thrace

*Prenolepis nitens* (Mayr, 1853)

Records in Greek Thrace: 5, 8, 51, 69, 70

Distribution in Greece and neighbouring regions: East Aegean Is., Epirus, Ionian Is., Macedonia, Sterea Ellas, Peloponnese, Thessaly; Bulgarian Thrace, Turkish Thrace

*Solenopsis fugax* (Latreille, 1798)

Records in Greek Thrace: 1

Distribution in Greece and neighbouring regions: Macedonia; Bulgarian Thrace, Turkish Thrace

Notes: See notes under S. cf. lusitanica.

**Figure 8.**
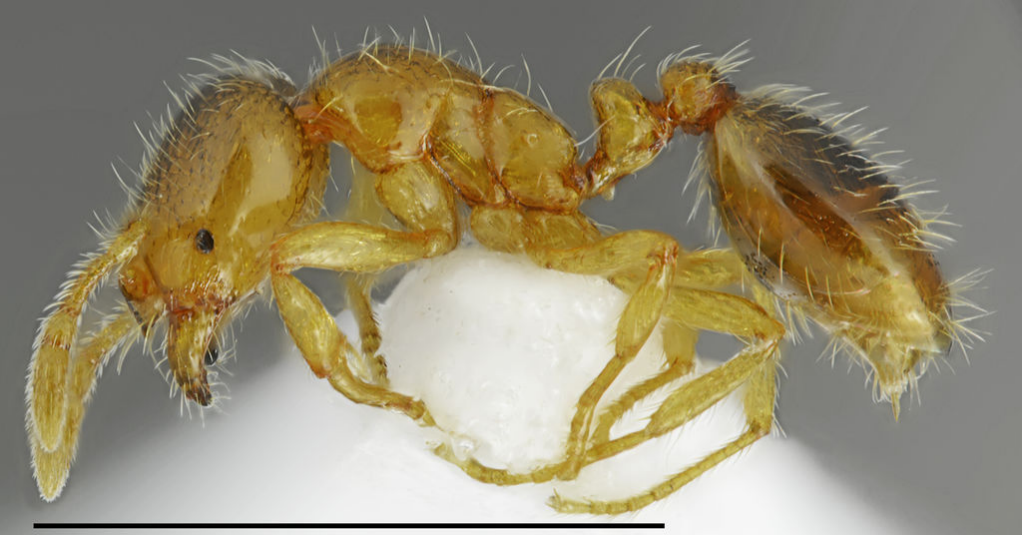
Solenopsis cf. lusitanica worker (specimen code: CASENT0763861). Lateral view of the body (scale bar = 1mm).

Solenopsis cf. lusitanica (Fig. 8​)

Records in Greek Thrace: 8, 9, 18, 22, 31, 36, 39, 41, 42, 44, 46, 47, 60, 61, 62, 65, 67, 69, 71

Distribution in Greece and neighbouring regions: Crete, Cyclades, Dodecanese, East Aegean Is., Ionian Is., Macedonia, Peloponnese, Thessaly

Notes: The status of most European species of genus *Solenopsis* in Europe requires an extensive revision. Galkowski et al. (2010) redescribed *Solenopsis fugax* and suggested that four distinct species groups occur in the territory of Europe and the Mediterranean area. They also suggested that several taxa proposed by Bernard (1950) are probably synonyms but they did not take any formal nomenclatorial decisions. In Thrace, we found samples belonging to at least two distinct morphospecies. One sample from the Rhodope Mountains (no. 1) appears to be true *S. fugax*. This species seems to be rare in Greece and we have only few samples from Macedonia in our collection. All other samples, characterized by shorter hairs on mesosoma and small gynes, belong to *Solenopsis lusitanica* group as proposed by Galkowski et al. (2010). Probably most of literature records of *S. fugax* from lowland, warm areas and regions outside Macedonia and Thrace concern taxa of *S. lusitanica* group. According to Galkowski et al. 2010, the group comprises three taxa described from the western part of the Mediterranean Basin, but we cannot exclude the presence of other undescribed species in the eastern Mediterranean.

*Stigmatomma denticulatum* Roger, 1859

Records in Greek Thrace: 66

Distribution in Greece and neighbouring regions: Crete, Dodecanese, East Aegean Is., Epirus, Ionian Is., Peloponnese, Sterea Ellas; Turkish Thrace

*Tapinoma erraticum* (Latreille, 1798)

Records in Greek Thrace: 1, 3, 4, 9, 10, 13, 25, 43, 51, 55, 56, 57, 58, 67, 68, 69, 70, 71

Distribution in Greece and neighbouring regions: Crete, Dodecanese, East Aegean Is., Epirus, Ionian Is., Macedonia, Peloponnese, Sterea Ellas, Thessaly; Bulgarian Thrace, Turkish Thrace

*Tapinoma simrothi* Krausse, 1911

Records in Greek Thrace: 48

Distribution in Greece and neighbouring regions: Crete, Cyclades, Dodecanese, East Aegean Is., Ionian Is., Macedonia, Peloponnese, Sterea Ellas, Thessaly; Turkish Thrace

**Figure 9.**
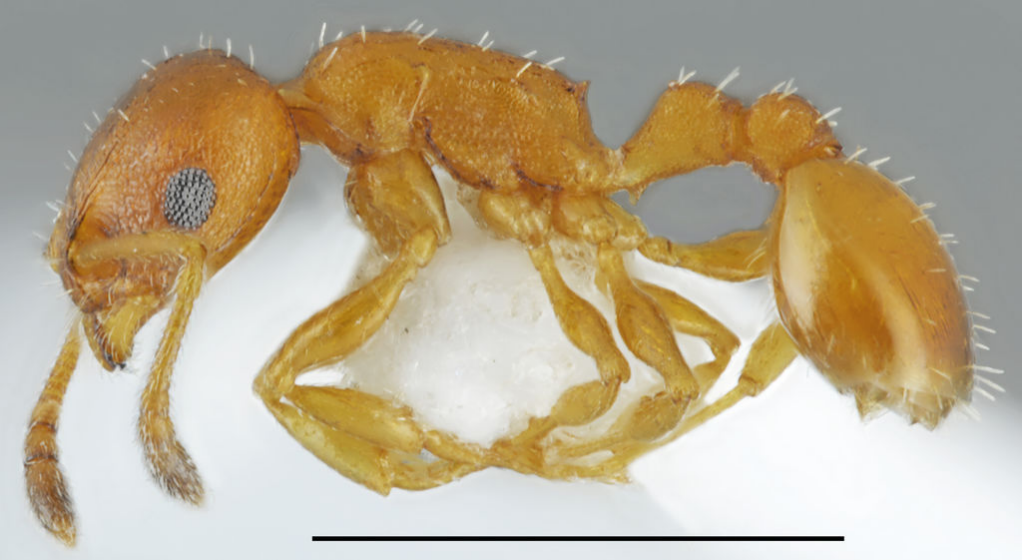
*Temnothorax aeolius* worker (specimen code: CASENT0763862). Lateral view of the body (scale bar = 1mm).

*Temnothorax aeolius* (Forel, 1911) (Fig. 9​)

Records in Greek Thrace: 10

Distribution in Greece and neighbouring regions: Dodecanese, East Aegean Is.

*Temnothorax affinis* (Mayr, 1855)

Records in Greek Thrace: 8, 43

Distribution in Greece and neighbouring regions: Cyclades, East Aegean Is., Macedonia, Sterea Ellas; Bulgarian Thrace, Turkish Thrace

**Figure 10.**
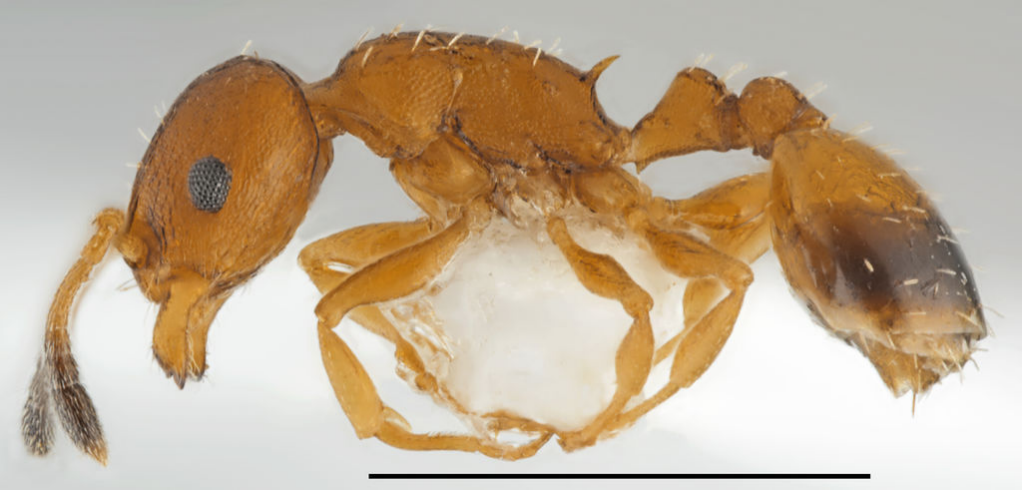
Temnothorax cf. affinis worker (specimen code: CASENT0763863). Lateral view of the body (scale bar = 1mm).

Temnothorax cf. affinis (Fig. 10)

Records in Greek Thrace: 43

Notes: Our sample named here as Temnothorax cf. affinis slightly differs in sculpture of head and lighter coloration from Central European population of *T. affinis* and those from locality 8 and one other sample from the locality 43. In our opinion Balkan populations of *T. affinis* need a revision based on detailed morphometric studies.

*Temnothorax bulgaricus* (Forel, 1892)

Records in Greek Thrace: 25, 43

Distribution in Greece and neighbouring regions: Dodecanese, East Aegean Is., Ionian Is., Peloponnese; Bulgarian Thrace, Turkish Thrace

Notes: See notes under Temnothorax cf. bulgaricus.

**Figure 11.**
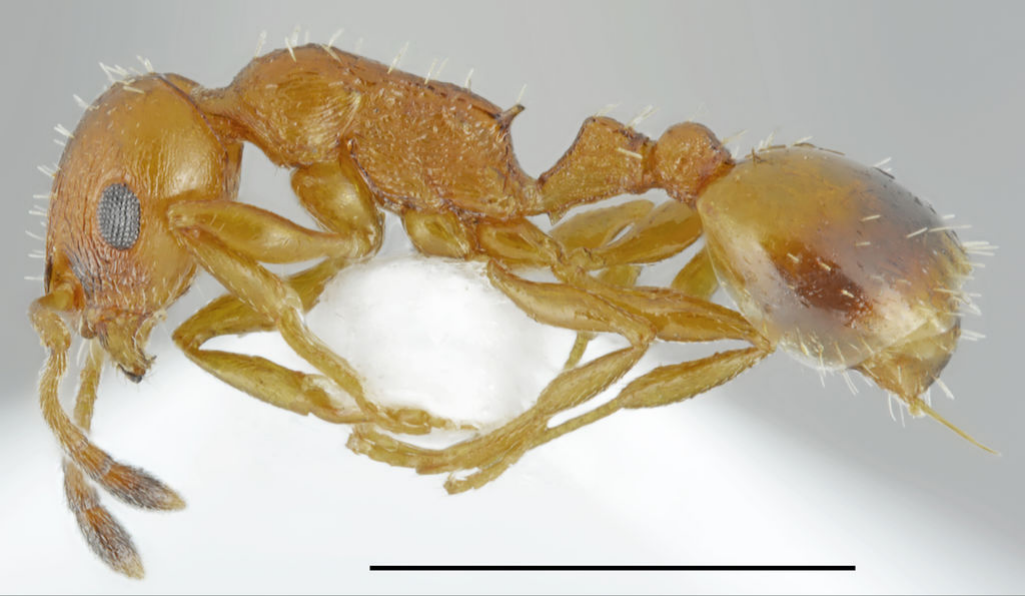
Temnothorax cf. bulgaricus worker (specimen code: CASENT0763864). Lateral view of the body (scale bar = 1mm).

Temnothorax cf. bulgaricus (Fig. 11)

Records in Greek Thrace: 10, 71

Notes: Balkan and Turkish populations of *Temnothorax bulgaricus* group need a revision based on detailed morphometric studies. Recently collected material suggests a big diversity of taxa within this group. Samples from the localities 25 and 43 well agree with studied types of *T. bulgaricus* while samples from the localities 10 and 71 belong to another species of this group. Therefore, prior to the revision of all taxa in this group, proper identification is impossible.

*Temnothorax crasecundus* Seifert & Csősz, 2015

Records in Greek Thrace: 55, 56, 59, 71

Distribution in Greece and neighbouring regions: Macedonia, Peloponnese; Bulgarian Thrace, Turkish Thrace

*Temnothorax exilis* (Emery, 1869)

Records in Greek Thrace: 6, 30, 33, 38, 68

Distribution in Greece and neighbouring regions: Crete, Dodecanese, East Aegean Is., Ionian Is., Macedonia, Peloponnese, Sterea Ellas; Turkish Thrace

**Figure 12.**
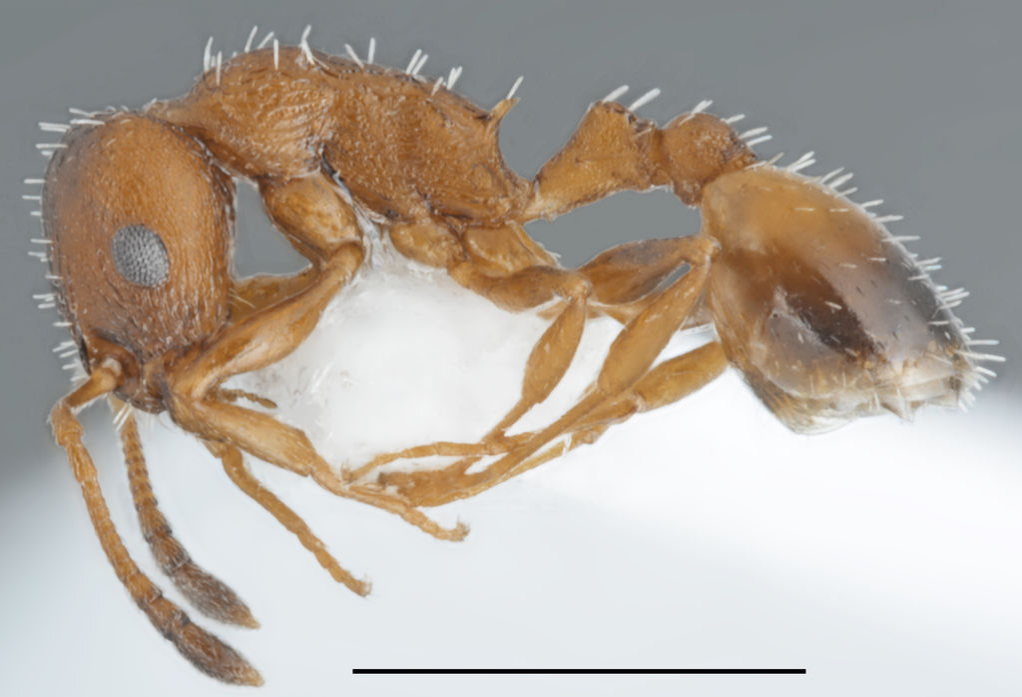
Temnothorax cf. graecus worker (specimen code: CASENT0763865). Lateral view of the body (scale bar = 1mm).

Temnothorax cf. graecus (Fig. 12​)

Records in Greek Thrace: 38, 43

Distribution in Greece and neighbouring regions: Crete, Cyclades, Dodecanese, Ionian Is., Macedonia, Peloponnese, Sterea Ellas, Thessaly; Turkish Thrace

Notes: Balkan and Turkish populations of *Temnothorax graecus* need a revision based on detailed morphometric studies. Our collected material from the Balkan Peninsula suggests that this taxon comprises several cryptic species.

**Figure 13.**
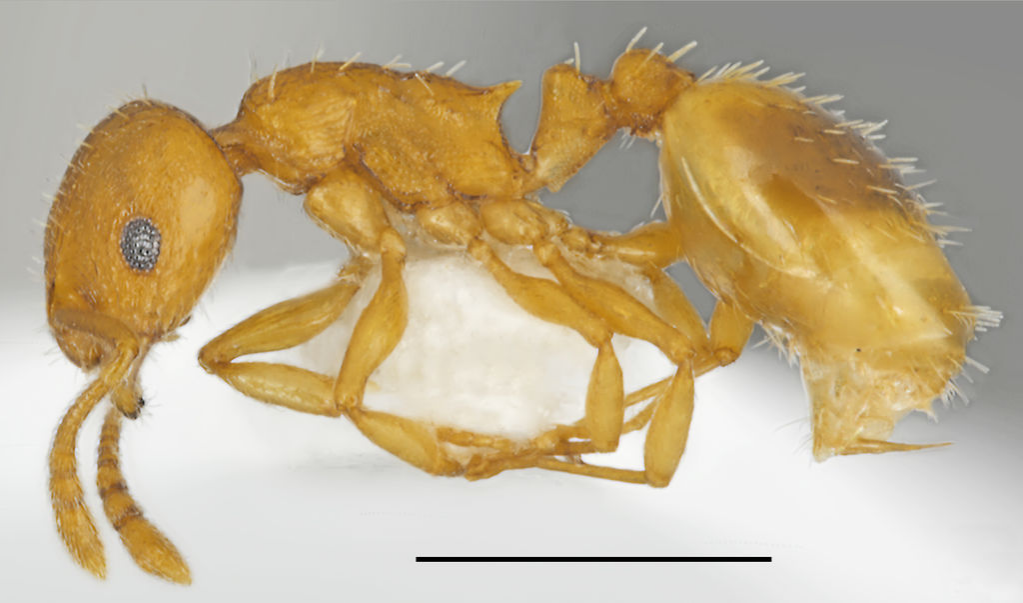
*Temnothorax helenae* worker (specimen code: CASENT0763866). Lateral view of the body (scale bar = 1mm).

*Temnothorax helenae* Csősz, Heinze & Mikó, 2015 (Fig. 13)

Records in Greek Thrace: 60, 63

Distribution in Greece and neighbouring regions: Crete, Macedonia, Sterea Ellas, Peloponnese, Thessaly; Bulgarian Thrace, Turkish Thrace

Temnothorax cf. interruptus

Records in Greek Thrace: 9, 37, 61, 64, 68

Distribution in Greece and neighbouring regions: Ionian Is., Macedonia, Thessaly; Bulgarian Thrace, Turkish Thrace

Notes: Recent studies suggest that true *T. interruptus* (Schenck, 1852) does not occur in Greece. All Greek populations belong to one or more undescribed taxa of the *T. interruptus* complex (Csősz et al. in preparation).

*Temnothorax lichtensteini* (Bondroit, 1918)

Records in Greek Thrace: 18, 65

Distribution in Greece and neighbouring regions: Epirus, Macedonia, Sterea Ellas, Thessaly; Turkish Thrace

*Temnothorax mediterraneus* Ward, Brady, Fisher & Schultz, 2015

Records in Greek Thrace: 8

Distribution in Greece and neighbouring regions: Crete, Macedonia

*Temnothorax nigriceps* (Mayr, 1855)

Records in Greek Thrace: 58

Distribution in Greece and neighbouring regions: Ionian Is., Macedonia, Peloponnese; Turkish Thrace

*Temnothorax parvulus* (Schenck, 1852)

Records in Greek Thrace: 26, 57, 59, 60

Distribution in Greece and neighbouring regions: Ionian Is., Macedonia; Bulgarian Thrace, Turkish Thrace

*Temnothorax recedens* (Nylander, 1856)

Records in Greek Thrace: 6, 8, 17, 20, 31, 36, 39, 41, 43, 44, 46, 47, 50

Distribution in Greece and neighbouring regions: Crete, Dodecanese, East Aegean Is., Ionian Is., Macedonia, Peloponnese, Sterea Ellas, Thessaly; Bulgarian Thrace, Turkish Thrace

*Temnothorax semiruber* (André, 1881)

Records in Greek Thrace: 10, 26, 31, 37, 69, 71

Distribution in Greece and neighbouring regions: Crete, Cyclades, Dodecanese, East Aegean Is., Ionian Is., Macedonia, Peloponnese, Sterea Ellas, Thessaly; Bulgarian Thrace, Turkish Thrace

**Figure 14.**
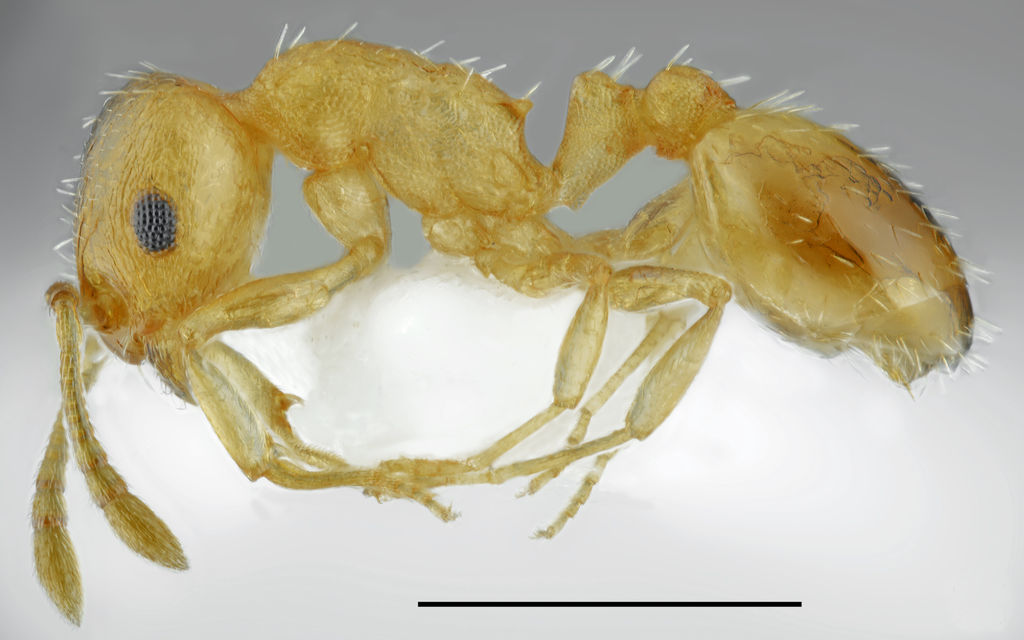
*Temnothorax subtilis* worker (specimen code: CASENT0763867). Lateral view of the body (scale bar = 1mm).

*Temnothorax subtilis* Csősz, Heinze & Mikó, 2015 (Fig. 14)

Records in Greek Thrace: 10, 60, 70

Distribution in Greece and neighbouring regions: Crete, Thessaly

*Temnothorax tergestinus* (Finzi, 1928)

Records in Greek Thrace: 5, 53

Distribution in Greece and neighbouring regions: Epirus, Macedonia, Thessaly; Bulgarian Thrace

Temnothorax cf. tuberum sp. 1 and sp. 2

Records in Greek Thrace: 1, 43

Distribution in Greece and neighbouring regions: Crete, East Aegean Is., Ionian Is., Macedonia, Thessaly; Turkish Thrace

Notes: Greek taxa belonging to *Temnothorax tuberum* group need revision based on detailed morphometric studies. Our material from various parts of Greece suggests that in this area occur more than one species related to *T. tuberum* (Fabricius, 1775). Specimens collected in Thrace appear to belong to two closely related species.

*Temnothorax turcicus* (Santschi, 1934)

Records in Greek Thrace: 71

Distribution in Greece and neighbouring regions: Macedonia, Thessaly; Bulgarian Thrace, Turkish Thrace

Temnothorax cf. unifasciatus sp. 1 and sp. 2

Records in Greek Thrace: 8, 18, 25, 26, 50, 52, 56, 57, 60, 61, 63, 66

Distribution in Greece and neighbouring regions: East Aegean Is., Epirus, Ionian Is., Macedonia, Thessaly; Turkish Thrace

Notes: Greek taxa belonging to *Temnothorax unifasciatus* group need revision based on detailed morphometric studies. Our material from various parts of Greece suggests that in this area occur at least two species related to *T. unifasciatus* (Latreille, 1798). Also, in our material from Thrace we have identified two morphospecies of this group.

*Tetramorium atratulus* (Schenck, 1852)

Records in Greek Thrace: 47

Distribution in Greece and neighbouring regions: Macedonia; Turkish Thrace

Tetramorium cf. caespitum sp. 1 and sp. 2

Records in Greek Thrace: 2, 6, 7, 10, 22, 33, 35, 42, 45, 46, 47, 48, 49, 59, 71

Distribution in Greece and neighbouring regions: Crete, Cyclades, Dodecanese, East Aegean Is., Epirus, Ionian Is., Macedonia, Peloponnese, Sterea Ellas, Thessaly; Bulgarian Thrace, Turkish Thrace

Notes: Molecular and morphometric studies suggest occurrence of at least nine species of *Tetramorium caespitum*/*impurum* complex in Europe and the Mediterranean area, but so far only five were named formally (Csősz and Markó 2004, Csősz et al. 2014, Schlick-Steiner et al. 2006a, Steiner et al. 2010). Our samples from Thrace belong to two named species (*T. hungaricum* and *T. impurum*) and two not formally described morphospecies we list here as T. cf. caespitum sp. 1 and sp. 2.

*Tetramorium chefketi* Forel, 1911

Records in Greek Thrace: 5, 22, 27, 35, 49, 51, 65, 67, 68, 70, 71

Distribution in Greece and neighbouring regions: Crete, East Aegean Is., Ionian Is., Macedonia, Thessaly; Bulgarian Thrace, Turkish Thrace

Tetramorium cf. davidi

Records in Greek Thrace: 25

Distribution in Greece and neighbouring regions: Dodecanese; Turkish Thrace

Notes: This sample belongs to a species group with head costulae diverging on occipital part of head and needs a revision. Our material from the eastern part of the Mediterranean suggests the occurrence of at least two distinct morphospecies.

Tetramorium cf. depressum sp. 1

Records in Greek Thrace: 12, 68

Notes: See notes under Tetramorium cf. semilaeve.

Tetramorium cf. depressum sp. 2

Records in Greek Thrace: 71

Notes: See notes under Tetramorium cf. semilaeve.

*Tetramorium ferox* Ruzsky, 1903

Records in Greek Thrace: 27, 42, 46, 72

Distribution in Greece and neighbouring regions: Crete, Cyclades, Dodecanese, East Aegean Is., Ionian Is., Macedonia; Bulgarian Thrace, Turkish Thrace

**Figure 15.**
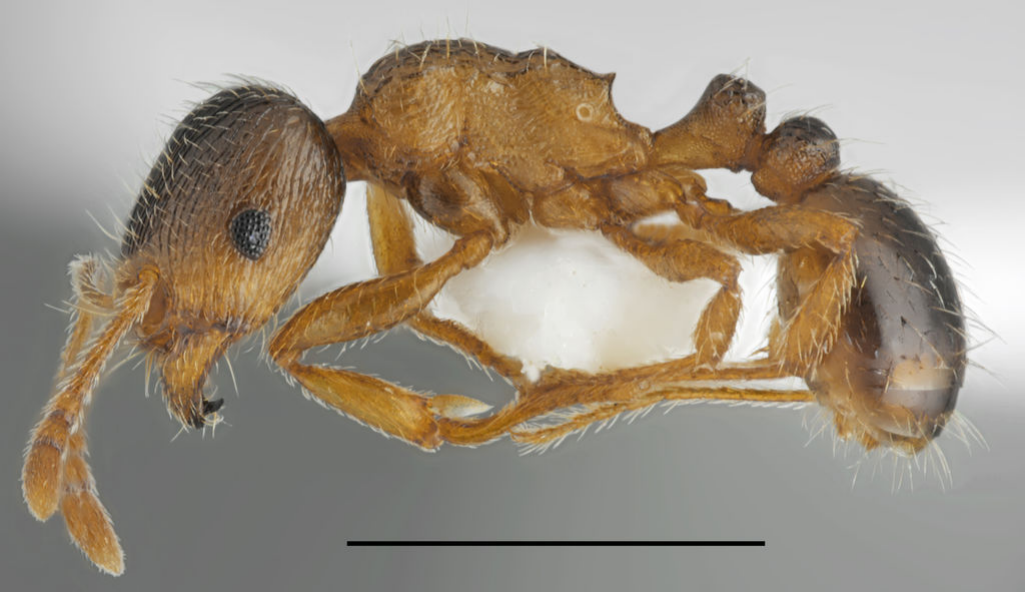
Tetramorium cf. flavidulum worker (specimen code: CASENT0763868). Lateral view of the body (scale bar = 1mm).

Tetramorium cf. flavidulum (Fig. 15)

Records in Greek Thrace: 56

Distribution in Greece and neighbouring regions: East Aegean Is., Dodecanese, Macedonia

Notes: In the eastern part of Mediterranean Basin, *Tetramorium flavidulum* group is represented by several morphospecies with centre of diversity in Anatolian Turkey (our unpublished data). Our material from Greece suggests the occurrence of at least three distinct species in this country. Male genitalia, petiole and postpetiole sculpture of the members of this group are very similar to those of the species of *T. chefketi* group revised by Csősz et al. 2007. However, all investigated specimens are devoid of microsculpture on the first gastral tergite, characteristic of the *T. chefketi* group. Proper identification of the sample from Thrace is impossible prior to the revision of all taxa of *T. flaviulum* group.

**Figure 16.**
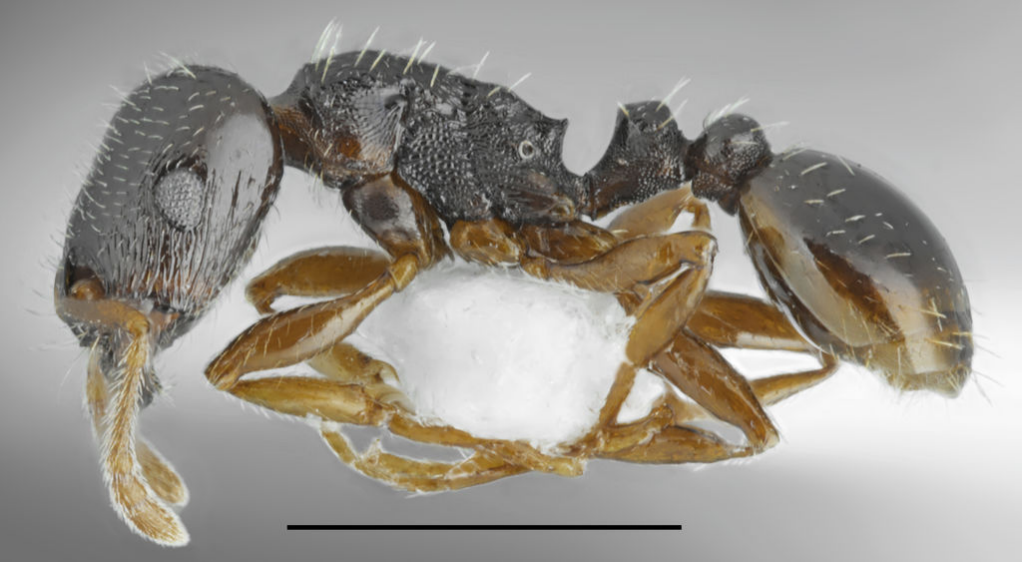
*Tetramorium hippocratis* worker (specimen code: CASENT0763870). Lateral view of the body (scale bar = 1mm).

*Tetramorium hippocratis* Agosti & Collingwood, 1987 (Fig. 16)

Records in Greek Thrace: 39

Distribution in Greece and neighbouring regions: Dodecanese, East Aegean Is.; Turkish Thrace

Notes: See notes under Tetramorium cf. semilaeve.

*Tetramorium hungaricum* Röszler, 1935

Records in Greek Thrace: 3, 25, 26, 58, 71,

Distribution in Greece and neighbouring regions: Macedonia, Thessaly; Bulgarian Thrace, Turkish Thrace

*Tetramorium impurum* (Förster, 1850)

Records in Greek Thrace: 1

Distribution in Greece and neighbouring regions: Macedonia; Turkish Thrace

*Tetramorium moravicum* Kratochvil, 1941

Records in Greek Thrace: 3, 4, 5, 7, 26, 55, 56, 57, 58, 59

Distribution in Greece and neighbouring regions: Crete, Epirus, Macedonia, Peloponnese, Thessaly; Bulgarian Thrace, Turkish Thrace

**Figure 17.**
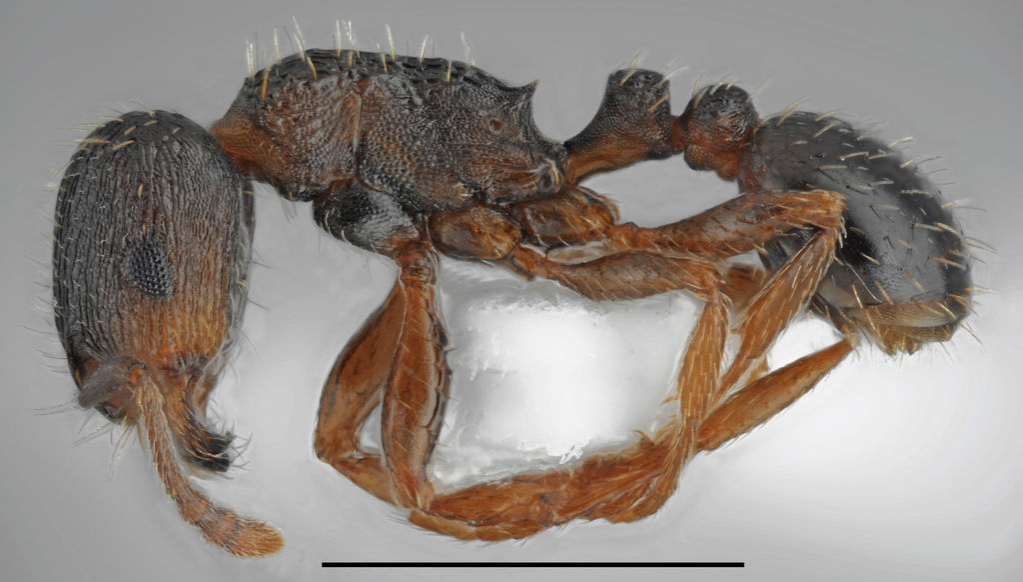
*Tetramorium rhodium* worker (specimen code: CASENT0763871). Lateral view of the body (scale bar = 1mm).

*Tetramorium rhodium* Emery, 1924 (Fig. 17)

Records in Greek Thrace: 9

Distribution in Greece and neighbouring regions: Dodecanese, East Aegean Is.

**Figure 18.**
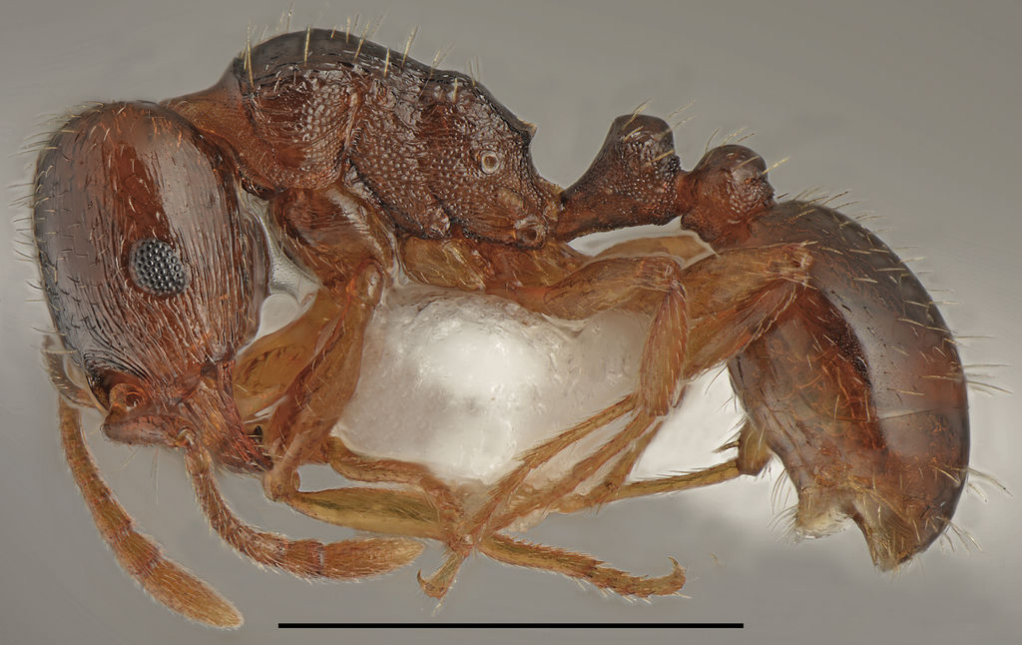
Tetramorium cf. semilaeve worker (specimen code: CASENT0763869). Lateral view of the body (scale bar = 1mm).

Tetramorium cf. semilaeve (Fig. 18)

Records in Greek Thrace: 9, 13, 17, 22, 29, 30, 32, 33, 35, 43, 48, 51, 68, 69; Legakis 2011

Distribution in Greece and neighbouring regions: Crete, Cyclades, Dodecanese, East Aegean Is., Epirus, Ionian Is., Macedonia, Peloponnese, Sterea Ellas, Thessaly; Turkish Thrace

Notes: *Tetramorium semilaeve* group from the Balkan Peninsula and Turkish Aegean regions is now under revision (Salata & Borowiec in preparation). We grouped samples with mostly reduced head sculpture under name *T. depressum* complex (*T. hippocratis* and at least three unnamed species) and taxa with more expressed head sculpture under *T. semilaeve* complex (at least two unnamed morphospecies and *T. galaticum* Menozzii, 1936). For proper identification of all taxa nest samples with males and gynes are required. In our material from Thrace we have three species from *T. depressum* complex (*T. hippocratis*, T. cf. depressum sp. 1 and T. cf. depressum sp. 2) and at least one species from *T. semilaeve* complex (T. cf. semilaeve). We managed to collect only one nest sample of *T. semilaeve* complex and it belongs to an undescribed species which seems to be widespread in Greece. Probably most literature records of *T. semilaeve* from Greece concern this undescribed taxon. True *T. semilaeve* André, 1883 is distributed only in western part of Mediterranean basin (Borowiec et al. 2015).

### Doubtful published records

Legakis 2011 listed five species from Thrace which are probably based on misidentification or misinterpretation.

*Aphaenogaster ovaticeps* (Emery, 1898)

Notes: This species, a member of *A. splendida* group, occurs only in Italy. Four other members of the group were recorded from Greece: *A. muelleriana* Wolf, 1915 from Epirus and Ionian Is., *A. splendida* (Roger, 1859) from Macedonia, Peloponnese and Sterea Ellas, *A. rugosoferruginea* Forel, 1889 endemic to Crete and *A. festae* Emery, 1915 from Dodecanese, East Aegean Is., Epirus, Macedonia and Thrace. Without voucher specimens it is impossible to determine which species was recorded by Legakis 2011.

*Lepisiota melas* (Emery, 1915)

Notes: In the key to Balkan ants (Agosti and Collingwood 1987a) *L. melas* is distingushed from *L. frauenfeldi* by the colouration of the mesosoma (alitrunk), being mainly or entirely reddish in *L. melas* and mainly or entirely dark in *L. frauenfeldi*. After examining photos of type specimens of both taxa (available in AntWeb https://www.antweb.org/specimen/CASENT0905146, https://www.antweb.org/specimen/CASENT0909884) it is evident that these species were misinterpreted and *L. melas* sensu Agosti & Collingwood = *L. frauenfeldi* while *L. frauenfeldi* sensu Agosti & Collingwood = *L. melas*. *Lepisiota melas* is southern species, in Greece occurs in the area south of Macedonia and Thrace while *L. frauenfeldi* is common in northern part of the country. With great probability the record of *L. melas* from Thrace in Legakis 2011 refers to *L. frauenfeldi*.

*Messor caducus* (Motschulsky, 1839)

Notes: Arnol'di 1977 placed several populations from Transcaucasia, Turkey and Central Asia under the name *M. caducus*, and described several infraspecific taxa. The closest to Greek border described taxon is *M. caducus caucasicola* Arnoldi, 1977 with type locality in Transcaucasia. Specimens of this taxon were recently collected in southwestern Turkey (our unpublished data). In our collection we also have a new endemic species from Crete, belonging to *M. caducus* group. Since all known localities for taxa of *M. caducus* group are far from Greek Thrace, we assume the record in Legakis 2011 probably does not belong to this group but to a related *M. semirufus* group.

*Messor bouvieri* Bondroit, 1918

Notes: *M. bouvieri* is a western Mediterranean species distributed from Portugal to Italy and its occurrence in Greece is unlikely. Record in Legakis 2011 is probably based on misidentification of one of the species from the *M. semirufus* group.

*Tetramorium lucidulum* Menozzii, 1933

Notes: This species was misinterpreted in the key to Balkan ants (Agosti and Collingwood 1987a). True *T. lucidulum* is a distinct species of *T. semilaeve* group (*T. depressum* complex), well distinguished by extremely narrow frons. It was described from “Syrien, Kleinasien, Turkestan” by Emery 1909 under unavailable name Tetramorium caespitum punicum var. lucidula, and is most probably absent from Greece. At least four other species of *T. depressum* complex occur in Greece and it is impossible to conclude what is the identity of the species mentioned in Legakis 2011 under the name *T. lucidulum*. See also notes under T. cf. semilaeve.

## Discussion

Although Thracian ant fauna has been almost totally neglected thus far, we can consider this Greek region as relatively diverse. We collected 115 species, only 7 already mentioned in the checklist in Legakis 2011. Other five taxa mentioned in this checklist can be treated as doubtful records and they probably concern species found also by us, but we cannot specify their true identity without checking voucher specimens. Thus, in total we can confirm the presence of 115 ant species for Greek Thrace. Out of the 11 geographic regions of Greece, only Macedonia with 158 species has richer ant fauna, while Dodecanese, East Aegean Islands and Ionian Islands have similar number of registered species (Borowiec & Salata unpubl. data). One reason for high species richness in Greek Thrace is probably the geographic position of the region, which is situated between the Aegean Sea and the Rhodope Mountains. As the result, we can encounter here species which are otherwise more common in the southern Greek regions, islands of eastern part of Aegean Sea or Aegean part of Turkey (e.g. *Aphaenogaster festae*, *Camponotus kiesenwetteri*, *Camponotus samius*, *Cataglyphis viaticoides*, *Temnothorax helenae*) as well as species with more northern distribution that are rarely found in other parts of Greece (e.g. *Formica fusca*, *Formica pratensis*, *Formica rufa*, *Lasius fuliginosus*, *Lasius jensi*, *Lasius niger*, *Leptothorax acervorum*, *Myrmica hellenica*, *Myrmica lonae*, *Myrmica specioides*, *Tetramorium impurum*). Among the collected material, we have some particulary interesting species, as they have not been found in the continental part of Greece yet, namely *Cataglyphis viaticoides*, *Temnothorax aeolius*, Tetramorium cf. davidi, *Tetramorium hippocratis* and *Tetramorium rhodium*. *Temnothorax aeolius* and *Tetramorium rhodium* are also absent from the neighbouring regions of Bulgarian and Turkish Thrace. In the checklist of species, we listed some taxa under names that have not been mentioned in the literature for Greece, i.e. Messor cf. ebeninus, Messor cf. semirufus, Temnothorax cf. affinis, Temnothorax cf. bulgaricus, Tetramorium cf. depressum sp. 1, Tetramorium cf. depressum sp. 2. Since they belong to taxonomically problematic groups it is very possible that they have already been recorded under different names from other parts of the country. Many ant genera and species groups are taxonomically unresolved so we cannot give the exact names for several species from the checklist. Some of them probably have available specific or infraspecific names now trated as synonyms of different taxa, and some are probably taxa new to science.

Results of the present study and recent investigations from other parts of the country show great richness of Greek ant fauna. Based on the material we have collected from various parts of Greece and which includes many still unidentified taxa (Borowiec & Salata unpublished data), it is estimated that at least 320 ant species occur in the fauna of Greece, several of them new to science.

## Figures and Tables

**Figure 4a.**
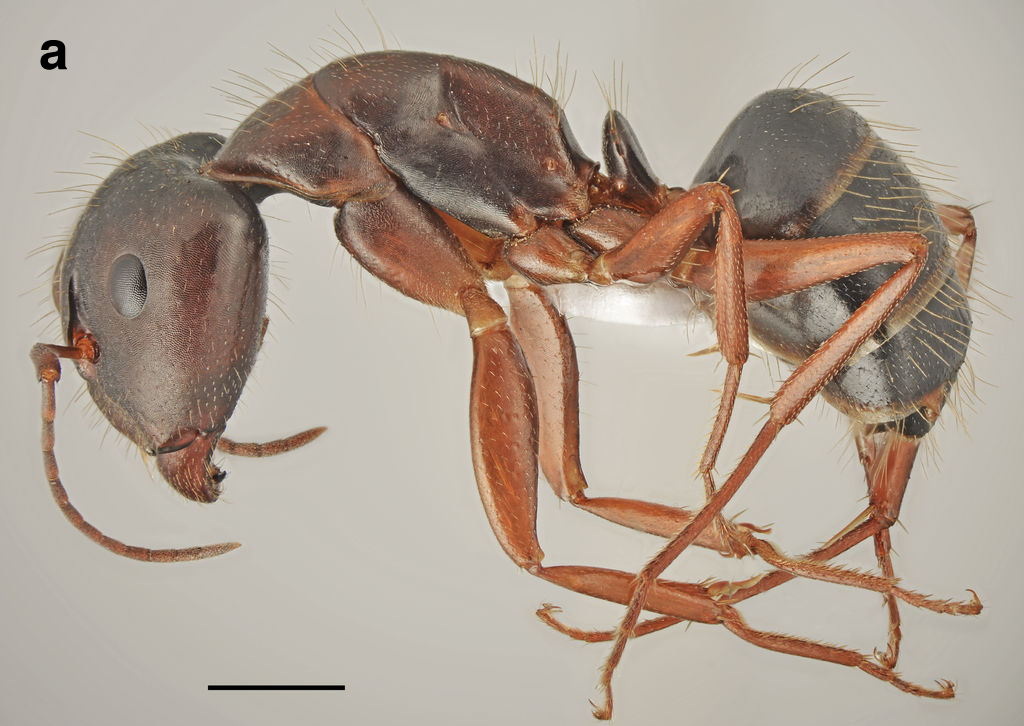
major worker (specimen code: CASENT0763855)

**Figure 4b.**
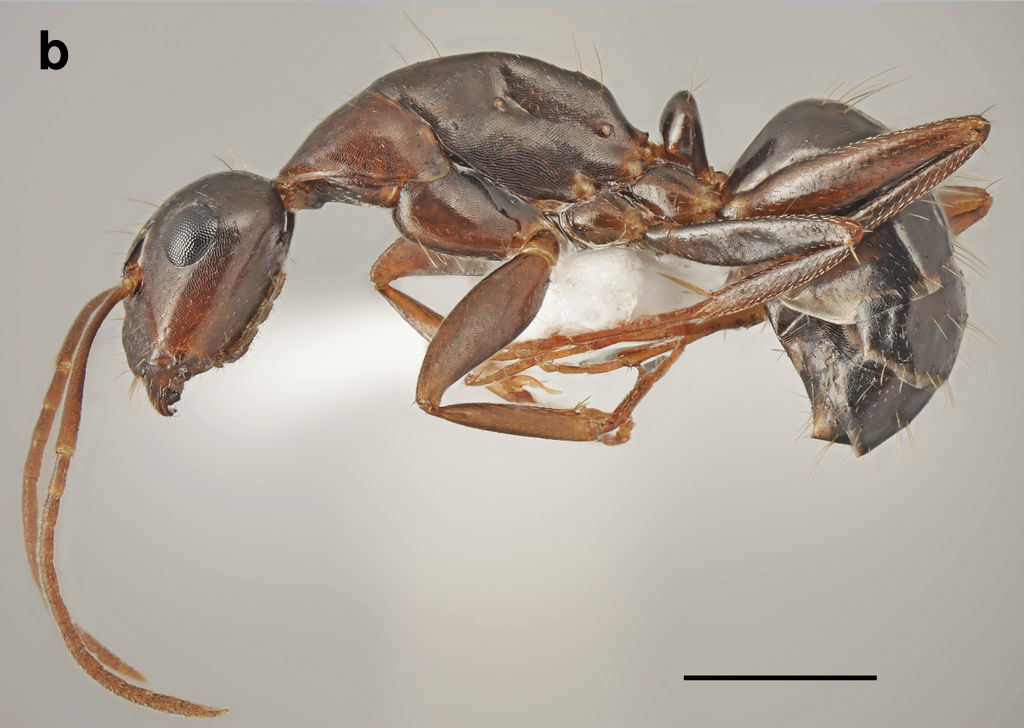
minor worker (specimen code: CASENT0763856)

**Figure 5a.**
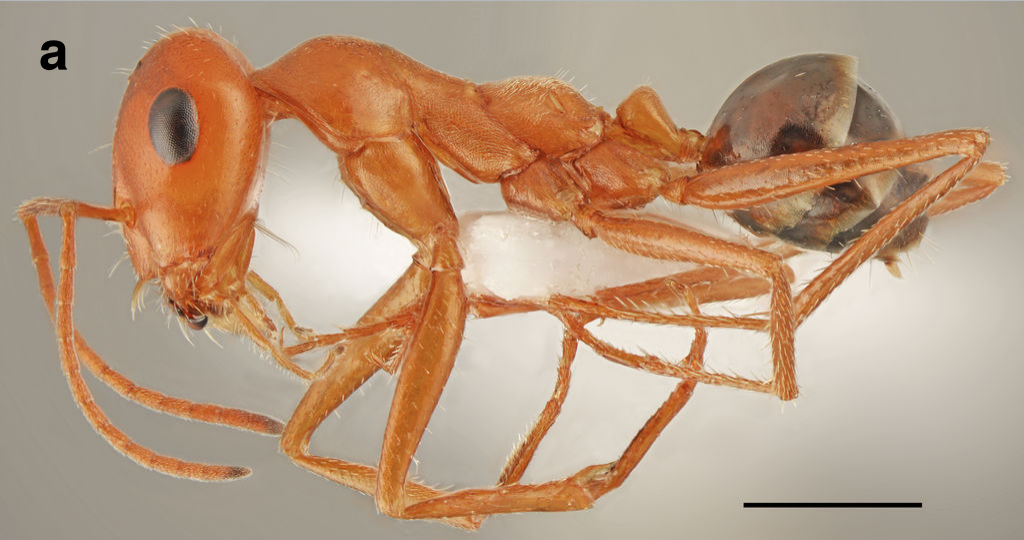
worker (specimen code: CASENT0763858)

**Figure 5b.**
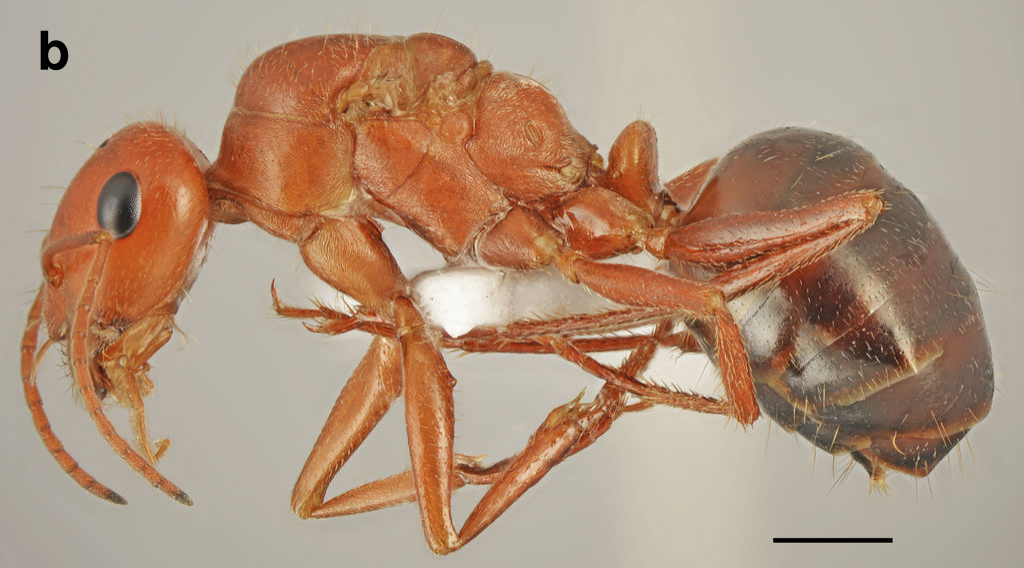
gyne (specimen code: CASENT0763857)

**Figure 7a.**
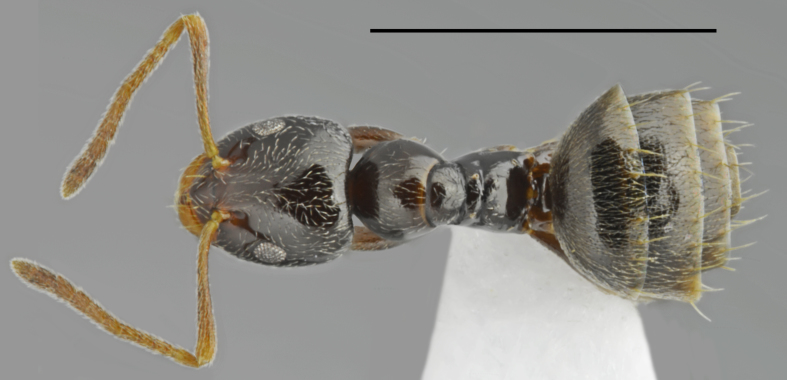
dorsal view of the body

**Figure 7b.**
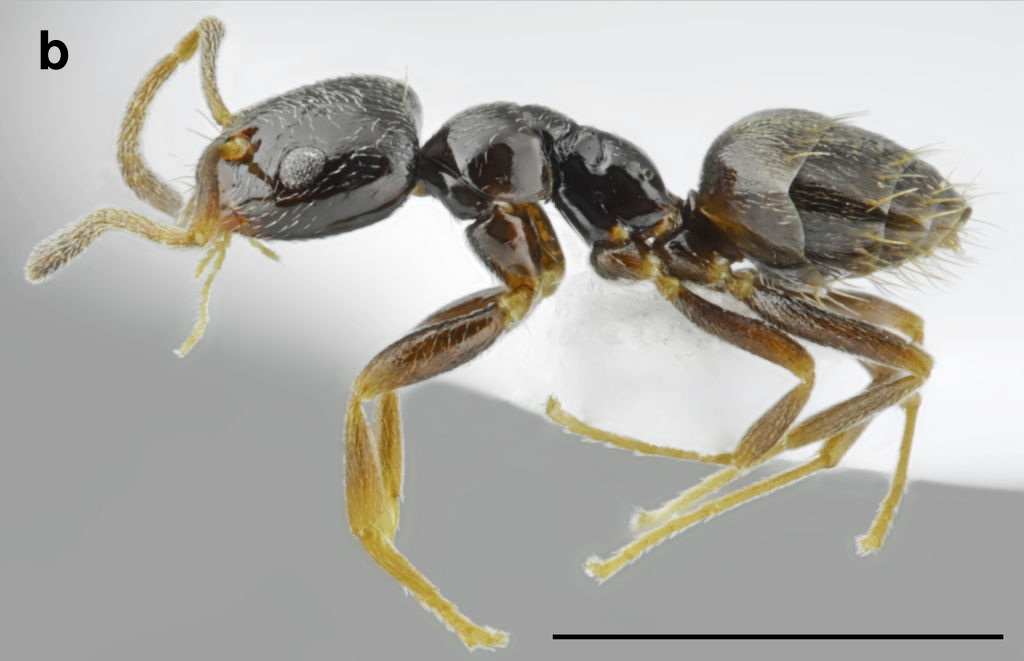
lateral view of the body

**Table 1.** Description of the localities in Greek Thrace where ants were sampled in 1999 and 2013 – 2015.

**Locality code**	**Locality**	**Municipality**	**Regional unit**	**Habitat**	**Coordinates**	**Altitude (m)**	**Date**	**Collector**
1	2 km S of Leivaditis	Xanthi	Xanthi		41.2874°N, 24.6651°E	1200	12.8.1999	E. Nikolakakis
2	3 km N of Ano Karyofyto	Xanthi	Xanthi	mountain grassland/ pasture with scrub and rocks	41.2865°N, 24.66617°E	1180	28.4.2014	G. Bračko
3	1.5 km NW of Dafnonas	Xanthi	Xanthi	grassland with some scrub and trees	41.228°N, 24.6605°E	220	28.4.2014	G. Bračko
4	2.5 km NE of Kallithea	Xanthi	Xanthi	scrub with some trees; deciduous forest	41.2595°N, 24.76183°E	1030	28.4.2014	G. Bračko
5	Komnina-Ano Livera road	Topeiros	Xanthi		41.1595°N, 24.6961°E	450	10.10.1999	E. Nikolakakis
6	1.5 km W of Galani	Topeiros	Xanthi	thermophilous rocky slope on limestone with scrub and some trees; parking area with some trees	41.0935°N, 24.75883°E	70	3.5.2014	G. Bračko
7	1.5 km N of Kirnos	Topeiros	Xanthi	field path with some trees	41.00133°N, 24.7752°E	20	27.4.2014	G. Bračko
8	2 km NW of Echinos	Myki	Xanthi	thermophilous deciduous forest on a stony ground	41.295°N, 24.96383°E	370	28.4.2014	G. Bračko
9	4.5 km SW of Nea Kessani	Abdera	Xanthi	grassland (partly wet); oak forest	41.0075°N, 25.02667°E	10	29.4.2014	G. Bračko
10	0.5 km W of Lagos	Abdera	Xanthi	pine forest on a wet ground	41.00683°N, 25.10783°E	5	29.4.2014	G. Bračko
11	Mesi	Komotini	Rodopi		40.983°N, 25.2095°E	20	3.7.2013	T.K. Amet
12	1.5 km N of Pandrosos	Komotini	Rodopi	dry scrub with some trees on a stony ground	41.19233°N, 25.44683°E	250	29.4.2014	G. Bračko
13	1.5 km W of Karidia	Komotini	Rodopi	grassland with some scrub	41.14322°N, 25.4202°E	110	29.4.2014	G. Bračko
14	Komotini	Komotini	Rodopi		41.12217°N, 25.4145°E	40	12.9.2013	T.K. Amet
15	Kikidio	Komotini	Rodopi		41.1075°N, 25.43283°E	50	11.9.2013	T.K. Amet
16	1 km SW of Drimi	Arriana	Rodopi		41.213°N, 25.56717°E	260	28.8.2013, 8.9.2013	Ş. Karabela
17	Drimi	Arriana	Rodopi	dry hills with scarce oak forest	41.21394°N, 25.5734°E	190	2.9.2015	L. Borowiec
18	1.3 km SW of Kato Drosini	Arriana	Rodopi	oak forest along dry stream valley	41.21515°N, 25.59189°E	260	2.9.2015	L. Borowiec
19	Kato Drosini	Arriana	Rodopi		41.22617°N, 25.59783°E	320	23.6.2013, 19.10.2013	T.K. Amet, Ş. Karabela
20	Kato Drosini-Drania road	Arriana	Rodopi	oak forest along dry stream valley	41.23172°N, 25.62033°E	250	2.9.2015	L. Borowiec
21	Neo Kallintiri	Arriana	Rodopi		41.14717°N, 25.572°E	70	19.-20.10.2013	Ş. Karabela
22	1 km N of Neo Kallintiri	Arriana	Rodopi	pasture along the river bank with several poplars	41.15683°N, 25.57605°E	70	2.9.2015	L. Borowiec
23	Dokos	Arriana	Rodopi		41.137°N, 25.597°E	100	24.9.2013	Ş. Karabela
24	Drosia	Arriana	Rodopi		41.16217°N, 25.63667°E	260	22.8.2013, 5.9.2013	T.K. Amet
25	4 km NE of Plagia	Arriana	Rodopi	open oak forest	41.157°N, 25.7595°E	630	30.4.2014	G. Bračko
26	2.5 km W of Kampos	Arriana	Rodopi	grassy slope with some scrub and trees	41.18717°N, 25.84117°E	840	30.4.2014	G. Bračko
27	Vragia	Arriana	Rodopi		41.09083°N, 25.55633°E	30	6.-8.9.2013, 15.9.2013	T.K. Amet
28	Archontika	Arriana	Rodopi		41.0725°N, 25.54167°E	30	5.9.2013	T.K. Amet
29	Salmoni	Maroneia-Sapes	Rodopi		41.00467°N, 25.5345°E	80	22.9.2013	M. Gazios-manpaşa
30	near Strimi	Maroneia-Sapes	Rodopi	dry stream valley with *Platanus* forest and mossy rocks	40.97329°N, 25.54116°E	170	5.9.2015	L. Borowiec
31	Krovili-Maroneia road	Maroneia-Sapes	Rodopi	pine forest with shale rocks	40.94286°N, 25.53441°E	210	5.9.2015	L. Borowiec
32	ancient Ismara, SE of Maroneia	Maroneia-Sapes	Rodopi	pastures with numerous stones and rocks inside the ruins of ancient settlements	40.86727°N, 25.53691°E	80	5.9.2015	L. Borowiec
33	Petrota	Maroneia-Sapes	Rodopi	northern slope of the hill with great rock	40.9013°N, 25.60546°E	220	28.8.2015	L. Borowiec
34	2.2 km N of Petrota	Maroneia-Sapes	Rodopi	coniferous forest with *Pinus nigra*	40.91982°N, 25.61517°E	130	29.8.2015	L. Borowiec
35	3.8 km W of Mesimvria	Maroneia-Sapes	Rodopi	dry pastures; dry stream valley	40.86628°N, 25.63135°E	10	28.8.2015	L. Borowiec
36	near Avra	Alexand-roupoli	Evros	dry oak forest with limestone rocks; dry stream valley	40.92227°N, 25.67666°E	250	28.8.2015	L. Borowiec
37	Dikella-Avra road	Alexand-roupoli	Evros	dry oak forest with limestone rocks	40.90827°N, 25.68754°E	230	28.8.2015	L. Borowiec
38	2 km N of Makri	Alexand-roupoli	Evros	scrubby slope on a stony ground	40.8725°N, 25.74183°E	250	2.5.2014	G. Bračko
39	Alexandroupoli-Kirki road (loc. 1)	Alexand-roupoli	Evros	oak forest edge	40.94409°N, 25.77836°E	350	29.8.2015	L. Borowiec
40	Alexandroupoli-Kirki road (loc. 2)	Alexand-roupoli	Evros	oak forest edge	40.92764°N, 25.80323°E	320	29.8.2015	L. Borowiec
41	Alexandroupoli-Kirki road (loc. 3)	Alexand-roupoli	Evros	oak forest	40.91697°N, 25.81271°E	220	29.8.2015	L. Borowiec
42	2.9 km E of Kirki	Alexand-roupoli	Evros	river valley with gravel; poplar forest	40.97157°N, 25.82591°E	160	29.8.2015	L. Borowiec
43	Alexandroupoli-Kirki road (loc. 4)	Alexand-roupoli	Evros	*Platanus* forest along a dry stream valley	40.90222°N, 25.83497°E	120	29.8.2015	L. Borowiec
44	1 km N of Palagia	Alexand-roupoli	Evros	pine forest	40.9194°N, 25.86687°E	330	6.9.2015	L. Borowiec
45	N of Avas (loc. 1)	Alexand-roupoli	Evros	rest area close to stream valley	40.97921°N, 25.91529°E	150	31.8.2015	L. Borowiec
46	N of Avas (loc. 2)	Alexand-roupoli	Evros	rest area close to road; oak forest with rocky walls	40.94644°N, 25.90533°E	100	31.8.2015	L. Borowiec
47	N of Avas (loc. 3)	Alexand-roupoli	Evros	*Platanus* forest along a river valley	40.94276°N, 25.91075°E	100	31.8.2015	L. Borowiec
48	Alexandroupoli, Hotel Plaza env.	Alexand-roupoli	Evros	ruderal sites in suburban area	40.84866°N, 25.84221°E	10	26.8.2015	L. Borowiec
49	Alexandroupoli, Port Area	Alexand-roupoli	Evros	urban park; ruderal sites in port area	40.84389°N, 25.8721°E	10	27.8.2015	L. Borowiec
50	1.6 km N of Loutros	Alexand-roupoli	Evros	rest area close to river with *Platanus* forest	40.89605°N, 26.05279°E	40	4.9.2015	L. Borowiec
51	0.5 km S of Itea	Alexand-roupoli	Evros	open oak forest; forest edge	40.96483°N, 26.18967°E	40	2.5.2014	G. Bračko
52	near Leptokaria	Alexand-roupoli	Evros	rest area close to roadside with mixed oak-beech forest	41.06553°N, 25.90852°E	750	3.9.2015	L. Borowiec
53	Aisymi-Leptokaria road (loc. 1)	Alexand-roupoli	Evros	rest area on the bank of oak forest	41.06121°N, 25.9122°E	750	30.8.2015	L. Borowiec
54	Aisymi-Leptokaria road (loc. 2)	Alexand-roupoli	Evros	thicket near a source of water intake	41.06043°N, 25.92451°E	690	30.8.2015	L. Borowiec
55	6.7 km NE of Leptokaria	Alexand-roupoli	Evros	fir forest on the banks with oak buffer zone	41.10967°N, 25.96282°E	910	30.8.2015	L. Borowiec
56	6.6 km NE of Leptokaria	Alexand-roupoli	Evros	mountain fir; pine forest	41.10698°N, 25.96444°E	930	3.9.2015	L. Borowiec
57	Leptokaria-Mega Dereio road (loc. 1)	Soufli	Evros	mountain pass of dwarf oak forest	41.1186°N, 25.94328°E	860	30.8.2015	L. Borowiec
58	Sapka, 13 km NE of Nea Santa	Soufli	Evros	mountain grassland with scrub and rocks	41.13383°N, 25.92267°E	900	30.4.2014	G. Bračko
59	Leptokaria-Mega Dereio road (loc. 2)	Soufli	Evros	mountain oak forest	41.15638°N, 25.92475°E	820	30.8.2015	L. Borowiec
60	Leptokaria-Mega Dereio road (loc. 3)	Soufli	Evros	mountain oak forest	41.16249°N, 25.95632°E	720	3.9.2015	L. Borowiec
61	4.4 km NW of Lefkimmi	Soufli	Evros	mixed pine and oak forest with mossy rocks	41.05035°N, 26.15935°E	280	4.9.2015	L. Borowiec
62	2.7 km NW of Lefkimmi	Soufli	Evros	dry stream valley inside pine forest on the site of the fire	41.03864°N, 26.16716°E	220	4.9.2015	L. Borowiec
63	Mt. Kapsalo, NW of Lefkimmi	Soufli	Evros	mountain oak forest with mossy rocks	41.09038°N, 26.13448°E	570	4.9.2015	L. Borowiec
64	4.9 km W of Dadia	Soufli	Evros	edge of an oak forest with mossy rocks	41.1206°N, 26.16635°E	180	1.9.2015	L. Borowiec
65	2.8 km W of Dadia	Soufli	Evros	oak forest	41.1241°N, 26.19078°E	140	1.9.2015	L. Borowiec
66	1.6 km NW of Dadia	Soufli	Evros	pine forest; dry stream valley	41.13659°N, 26.20799°E	40	1.9.2015	L. Borowiec
67	Dadia	Soufli	Evros	pine forest	41.12367°N, 26.21939°E	130	1.9.2015	L. Borowiec
68	0.5 km NW of Likofos	Soufli	Evros	rocks with moss and pines on a relatively wet ground	41.12283°N, 26.28533°E	40	2.5.2014	G. Bračko
69	2.5 km SE of Petrota	Orestiada	Evros	dry grassland with some scrub; forest edge	41.672°N, 26.15017°E	180	1.5.2014	G. Bračko, K. Kiran
70	2.5 km SE of Pentalofos	Orestiada	Evros	oak forest; forest edge	41.63117°N, 26.20583°E	220	1.5.2014	G. Bračko, K. Kiran
71	1 km NE of Rizia	Orestiada	Evros	grassland with some scrub and trees on a sandy ground; wet deciduous forest	41.63633°N, 26.43267°E	40	1.5.2014	G. Bračko, K. Kiran
72	Orestiada	Orestiada	Evros		41.50117°N, 26.52967°E	40	28.9.2013	M. Gazios-manpaşa
